# Cayman enables large-scale analysis of gut microbiome carbohydrate-active enzyme repertoires

**DOI:** 10.1038/s41564-026-02318-2

**Published:** 2026-04-24

**Authors:** Quinten R. Ducarmon, Nicolai Karcher, Samir Giri, Hanne L. P. Tytgat, Omar Delannoy-Bruno, Selin Pekel, Fabian Springer, Patrick Wörz, Christian Schudoma, Athanasios Typas, Georg Zeller

**Affiliations:** 1 Molecular Systems Biology Unit, European Molecular Biology Laboratory, Heidelberg, Germany; 2 Leiden University Center for Infectious Diseases, Leiden University Medical Center, Leiden, the Netherlands; 3 Nestlé Institute of Health Sciences, Nestlé Research, Société des Produits Nestlé S.A, Lausanne, Switzerland; 4 Laboratory of Microbiology, Wageningen University, Wageningen, the Netherlands; 5 Center for Microbiome Analyses and Therapeutics, Leiden University Medical Center, Leiden, the Netherlands

**Keywords:** Genome informatics, Microbial communities, Glycobiology

## Abstract

Carbohydrate-active enzymes (CAZymes) are crucial for digesting glycans, but tools for CAZyme profiling and interpretation of substrate preferences in microbiome data are lacking. Here we develop a CAZyme profiler called Cayman (Carbohydrate Active Enzymes Profiling of Metagenomes) and a hierarchical substrate annotation scheme for use with genomic or shotgun metagenomic datasets. Using these tools, we systematically surveyed CAZymes in human gut microorganisms (*n* = 107,683 genomes) and identified several putative mucin-foraging bacteria, including *Hungatella* and *Eisenbergiella* species, which were confirmed experimentally. We compared CAZymes in gut metagenomes (*n* = 3,960) from high-income settings versus low- and middle-income settings and found that low- and middle-income setting metagenomes are enriched in fibre-degrading CAZymes, while CAZyme richness is generally higher in high-income setting metagenomes. Additional analysis (*n* = 1,998) indicated that metagenomes of individuals with colorectal cancer are depleted in fibre-targeting and enriched in glycosaminoglycan-targeting CAZymes. Finally, we inferred CAZyme substrates from genomic co-localization of CAZyme domains. Cayman is broadly applicable and freely available from https://github.com/zellerlab/cayman.

## Abstract (web summary)

Cayman enables computational analysis of carbohydrate-active enzymes (CAZymes) in microbial community data. It revealed novel mucus-foraging gut microorganisms and differences in gut microbial CAZyme repertoires associated with lifestyle and disease.

## Main

Carbohydrate-active enzymes (CAZymes) act on glycans and glycoconjugates and are a key factor shaping the metabolic capacity of microbial communities^1^. The human gut microbiota can utilize an enormous diversity of diet-derived (complex) carbohydrates that are otherwise indigestible to the host—as the human genome only encodes 17 catabolic CAZymes^2^. While gut microorganisms greatly differ in their genomic CAZyme repertoires and preferred substrates, many gut microbial lineages possess CAZymes dedicated to degrading host-derived glycans, most importantly mucins lining the intestinal epithelium^2,3^. The *Bacteroides* genus is particularly CAZyme rich, and *Bacteroides* spp. can flexibly alternate between feeding on mucins and dietary fibre (DF) according to their availability in the gut^4^.

Gut microbial carbohydrate metabolism is crucial for host health. DFs can be fermented by bacteria into short-chain fatty acids, which promote epithelial barrier integrity and gut health^5,6^. Consequently, fibre-deprived diets can lead to erosion of the mucus layer as bacteria shift to mucus utilization, potentially weakening this barrier^3,4^. Compromised barrier integrity is a hallmark of inflammatory bowel disease and colorectal cancer (CRC), in which gut microbial CAZymes involved in degradation of host extracellular matrix and mucins are enriched, respectively^7,8^. Given the central role of the gut microbiome as a mediator of dietary effects on host health, understanding how a lifestyle typical for high-income settings (HIS; often referred to as ‘westernization’^9–11^) shapes the CAZyme repertoire can yield important insights into disease processes. Previous work has revealed drastic differences in human gut microbiome composition between HIS and low- and medium-income settings (LMIS; also called ‘non-Western’ settings) and substantiated the hypothesis that specific microorganisms and their functions, including CAZymes, ‘vanished’ in the process of ‘westernization’^12,13^. While the binary classification of lifestyles into HIS and LMIS is an oversimplification, the contrast has uncovered shifts in key microbiome functions, such as a relative increase in CAZymes facilitating mucin foraging in HIS individuals^14^. Despite the crucial roles of CAZymes for both microbial communities and their host, existing studies are limited to few isolate genomes and do not leverage contemporary (meta)genomic data resources^2,15,16^.

One underlying reason for the relative scarcity of metagenome-driven CAZyme studies is the lack of scalable and easy-to-use bioinformatics tools: despite the availability of the CAZy database (http://www.cazy.org), which provides a manually curated knowledge hub of CAZymes^17^, and the computational framework for automated CAZyme annotation (dbCAN)^18^, delineation and quantification of microbial CAZymes in human gut metagenomes is generally performed ad hoc owing to lack of open-source software for this task^8,14,19^. Furthermore, while CAZymes have been divided into the main classes of glycoside hydrolase (GH), polysaccharide lyase (PL), carbohydrate esterases (CE), glycosyl transferases (GT) and carbohydrate-binding module (CBM; which we include among CAZymes here despite their lack of catalytic activity), substrate information is more difficult to obtain. While substrate information has been collected in the CAZy database and through curation efforts of several groups^14,17^, there can be discrepant classifications.

Here, we developed an easy-to-use and freely available bioinformatics tool (https://github.com/zellerlab/cayman) (see ‘Code availability’ section) to identify and quantify the abundances of CAZymes in (gut) microbial communities from shotgun metagenomic data. We furthermore provide a substrate annotation scheme facilitating the interpretation of the resulting CAZyme profiles by grouping CAZyme families into biologically meaningful substrate groups, which we manually curated from the CAZy database^17^ and scientific literature. We applied these tools on large gut bacterial genome collections and metagenomic datasets demonstrating their utility for pinpointing bacterial species with specific substrate utilization patterns (for example, mucin foraging) and how substrate utilization can differ across host lifestyles and health states.

## Developing and curating a CAZyme substrate scheme

**Fig. 1:**
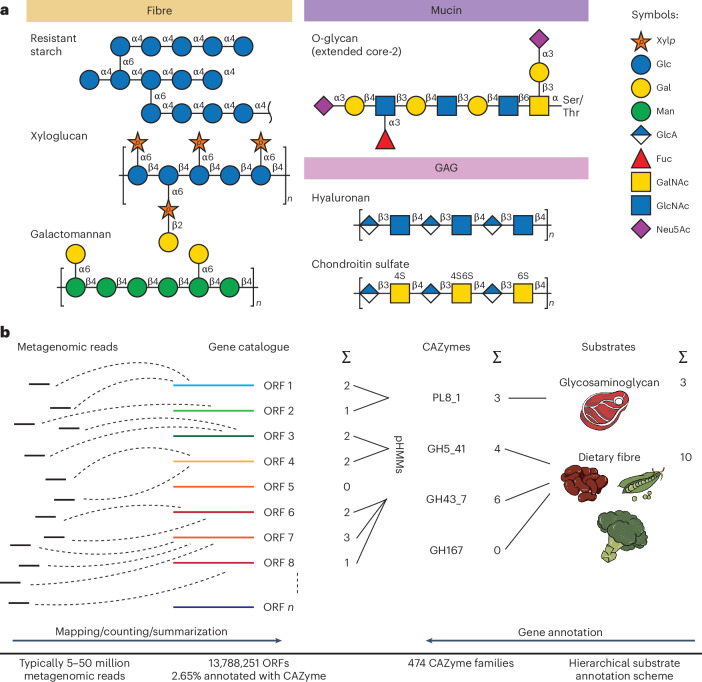
Cayman enables profiling of CAZymes and their glycan substrates from (meta-)genomic data. **a**, Examples of glycan structures and of their classification into high-level substrate categories. Xyl*p*, xylopyranose; Glc, glucose; galactose, Gal; Man, mannose; GlcA, glucuronic acid; Fuc, fucose; GalNAc, *N*-acetylgalactosamine; GlcNAc, *N*-acetylglucosamine; Neu5Ac, *N*-acetylneuraminic acid. **b**, Cayman is a computational tool for profiling CAZymes from metagenomes via quantification of CAZymes through the mapping of metagenomic sequencing reads to a gene catalogue. CAZyme abundances are then aggregated/grouped at different levels according to our curated substrate annotations.

Our understanding of CAZyme repertoires of microbial communities depends on detailed enzyme–substrate data, which also form the conceptual basis for automated analysis of CAZymes in metagenomic data. Here, we developed a comprehensive hierarchical substrate annotation scheme for CAZymes (Methods) with the aim to overcome limitations of previous efforts that were often incomplete and focused on GHs only^14,17^. Here, three authors manually curated available substrate information from http://www.cazy.org/ (ref. ^17^) and related scientific literature for all CAZyme (sub)families of the GH, PL and CBM categories represented in dbCAN2 version 9. Consensus substrate annotations were collected (Supplementary Table 1; Methods) and compared for agreement between Carbohydrate Active Enzymes Profiling of Metagenomes (Cayman) and dbCAN3 (Supplementary Fig. 1). We collected several layers of substrate annotation categories largely following previous recommendations for glycan classification^20^. Substrate annotations facilitate interpretation of downstream statistical analysis at various levels of granularity (Fig. 1a and Supplementary Table 2). For example, CBM2 binds cellulose, hemicellulose and chitin (annotation level 3), which are non-starch polysaccharides (NSPs; annotation level 2) of structural origin belonging to DFs (annotation level 1). It should be noted that, similar to CBM2, many CAZyme families are polyspecific, which is also reflected in our annotations with multiple possible substrate categories per CAZyme family.

## Cayman, a metagenomic CAZyme profiling tool

To be able to routinely quantify CAZymes in metagenomes, we developed a computational CAZyme profiling tool called Cayman. Its capabilities differ from dbCAN2/3 and other CAZy-related computational workflows in the following aspects (Supplementary Table 3): Instead of directly screening for fragments of CAZyme genes in (short) metagenomic sequencing reads, Cayman first maps reads to a gene catalogue, which represents a near-complete complement of microbial genes. Here, for analysing human faecal metagenomes, we relied on a consolidated, non-redundant human gut gene catalogue^21^, in which we annotated CAZymes using recalibrated profile Hidden Markov models (pHMMs; Methods). Functional profiling via gene-catalogue mapping provides faster and more accurate gene abundance estimates compared with annotating reads themselves^21,22^. Cayman finally calculates length- and sequencing depth-normalized CAZyme family abundances (Fig. 1b; Methods) from tallies of mapped reads that can be further summarized using the annotation categories introduced by our CAZyme substrate scheme to, for example, quantify mucin-utilization CAZymes in a given metagenome (Fig. 1b).

Cayman is broadly applicable to (meta-)genomic data from other microbial communities and freely available via GitHub (https://github.com/zellerlab/cayman).

## Genomic exploration of CAZyme repertoire in human gut microorganisms

**Fig. 2:**
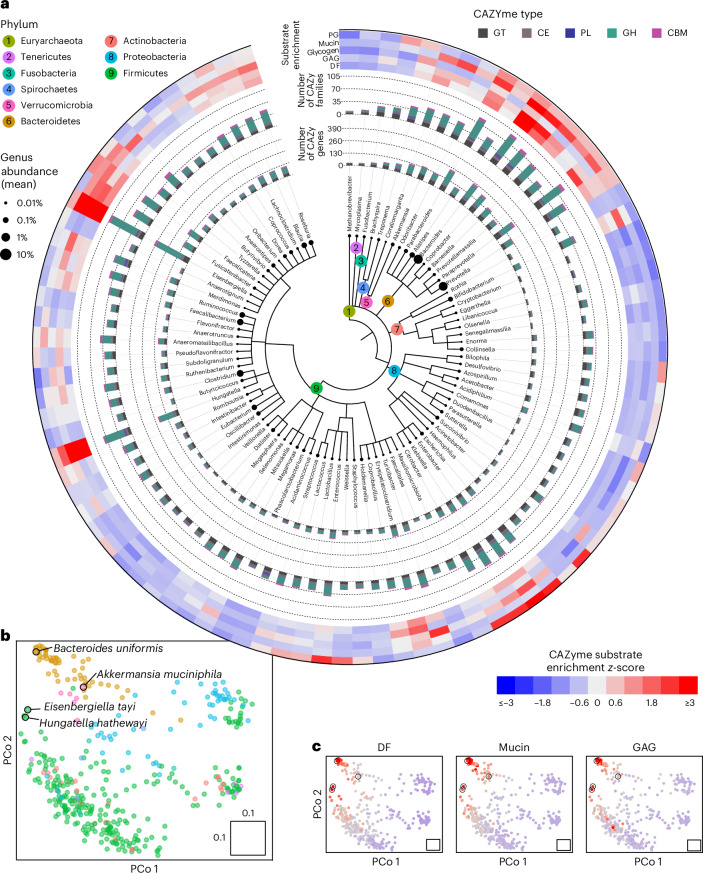
Genomic exploration of CAZymes in human gut bacteria. **a**, Taxonomic tree of 91 prevalent human gut microbial genera (based on *n* = 75,131 genomes) showing their mean relative abundance (leaf tips), number of CAZyme genes (inner coloured bar plot), number of unique CAZyme families (outer coloured bar plot) by type as well as CAZyme substrate enrichments (outermost heat map). Substrate enrichment was calculated as *z*-scores of the genus-wise mean total copy number of CAZymes annotated with a specific substrate class. **b**, Ordination plot depicting genomic CAZyme similarity between species-level mOTUs (phyla colour-coded as in **a**). CAZyme similarity between pairs of species was calculated as the Jaccard index of genomic absence or presence of CAZymes (Methods). Only species represented by more than three genomes are shown. PCo, principal coordinate. **c**, Same ordinations as shown in **b** but coloured by CAZyme substrate enrichment *z*-score (calculated in the same way as for genus-level enrichments shown in outer heat map in **a**). Source data

**Extended Data Fig. 1:**
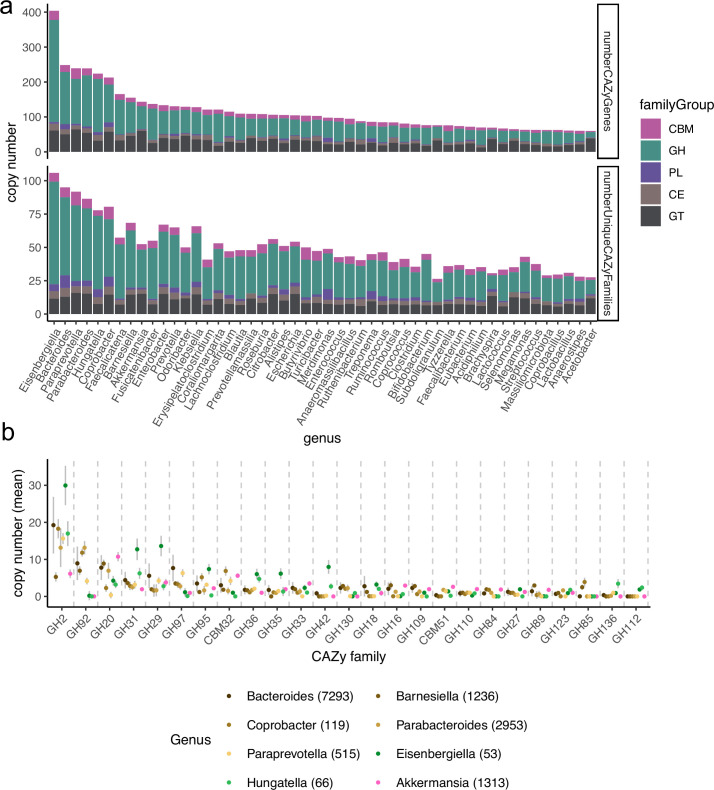
Copy number of CAZymes in bacterial genera. (**a**) Total number of genes with a CAZyme and number of unique CAZymes per genus. GH, PL, CE, GT and CBM. (**b**) Gene copy numbers of 25 mucin-related CAZy families from the eight genera with the highest mean copy number of mucin-metabolising CAZymes. The number in the parenthesis after the genus names correspond to the number of genomes considered for this analysis. Dots correspond to the mean of the data; Whiskers correspond to +/- 1 standard deviation of the data. Source data

Owing to recent advances in metagenome assembly, genomic resources of the human gut microbiome have substantially grown. To provide an updated view of the CAZyme repertoire of human gut microorganisms, we re-annotated genes from 107,683 high-quality metagenome-assembled genomes (MAGs) and isolate genomes^15^. Our analysis confirmed that the genomic potential for glycan metabolism strongly varies across taxonomy^2^ (Fig. 2a). In addition to confirming that key genera from the Bacteroidetes phylum, most prominently *Bacteroides* and *Parabacteroides*, have extensive CAZyme repertoires, our analysis also highlighted less studied ones, such as *Coprobacter* and *Paraprevotella*, to be particularly rich in CAZymes (213 genes from 80 families and 239 genes from 92 families, respectively). While Firmicutes generally showed lower CAZyme diversity (on average 77.2, s.d. 38.7, CAZymes per genome compared with an average of 181, s.d. 105, for Bacteroidetes), several genera from this phylum exhibited exceptionally rich CAZyme repertoires. Among these, *Hungatella* and *Eisenbergiella* stood out as distinctly rich in CAZymes (with 224 genes from 78 families and 404 genes from 106 families, respectively; Extended Data Fig. 1a).

We further compared the overall CAZyme repertoire of gut microbial taxa using ordination on the basis of pairwise similarity of CAZy family presence or absence (Methods). Species from the same phylum tended to cluster on the basis of their CAZyme repertoire. In addition, this ordination captures a gradient between small and large CAZyme repertoires (Fig. 2b,c). Accordingly, *Hungatella hathewayi, Eisenbergiella tayi* and the CAZyme-rich *Bacteroides uniformis* cluster together and farthest apart from gut species with substantially fewer CAZyme genes, such as members of Actinobacteria or Proteobacteria (Fig. 2b).

To investigate CAZyme substrate preferences among core gut bacterial genera, we leveraged our substrate annotations to compute enrichment scores of five major substrate classes: peptidoglycan (PG), mucin, glycogen, glycosaminoglycan (GAG) and DF (Fig. 2a; Methods). This analysis confirmed well-described mucin foragers such as *Akkermansia, Alistipes* and *Bacteroides* to possess many different mucin-targeting CAZyme genes. The poorly characterized *Barnesiellaceae* family included two genera, *Barnesiella* and *Coprobacter*, whose members were also strongly enriched in mucin-targeting CAZymes.

**Fig. 3:**
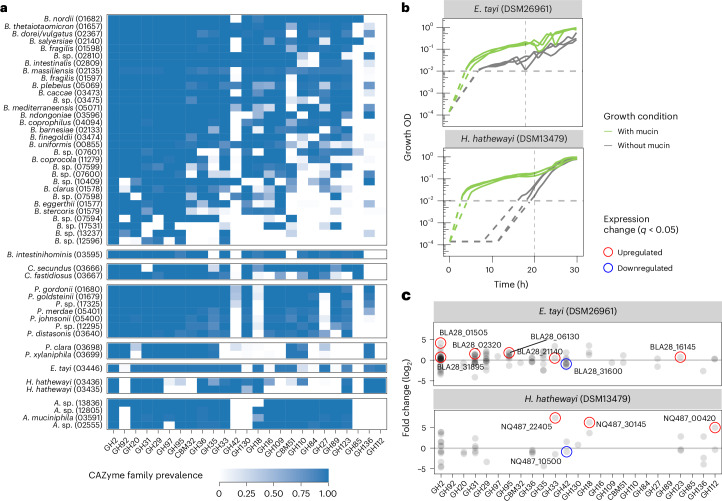
Gut microbial species and CAZymes targeting mucin as experimentally validated for *E. tayi* and *H. hathewayi.* **a**, Heat map showing the prevalences of 25 mucin-related CAZy families for species-level mOTUs from the eight genera with the highest mean copy number of mucin-metabolising CAZymes (genera from top to bottom: *Bacteroides*, *Barnesiella*, *Coprobacter*, *Parabacteroides*, *Paraprevotella*, *Eisenbergiella*, *Hungatella* and *Akkermansia*). Only species represented by more than three genomes are shown. **b**, Growth curves of *E. tayi* and *H. hathewayi* in rich medium (WCA) either with or without supplemental mucin (see the key) showing media-corrected OD (578 nm, log scale, three replicates). The limit of detection of the plate reader is at an OD_578_ of 0.01; the growth pattern below this is inferred (dashed lines) on the basis of the known inoculum and the fact that growth rate cannot be slower than what is measured above the limit of detection. The vertical dashed line corresponds to the sampling time for differential expression analysis in **c**. **c**, Differential gene expression analysis in the presence or absence of mucin. Source data

**Extended Data Fig. 2:**
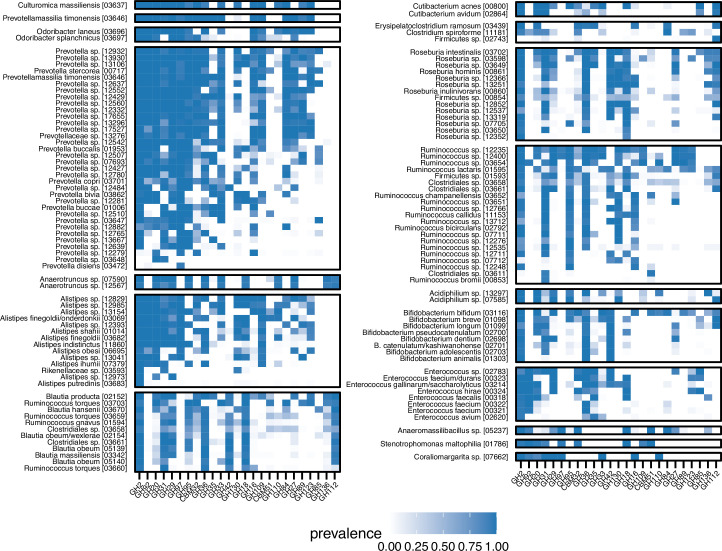
Heatmap of bacterial species and the genomic prevalence of mucin-metabolizing genes. Genomic prevalence of 25 mucin-related CAZyme families of the top mucin-related genera in the human gut. The display item is analogous to Fig. 3a, and genera were ranked using the same metric as in that display item, but instead of showing the top 8 genera, here we are showing the next 17 (such that together, the top 25 genera are shown). Source data

To resolve the mucin-targeting CAZyme repertoire at species resolution, we surveyed relevant CAZyme families across the eight genera exhibiting the strongest enrichment for mucin substrates. While this analysis revealed considerable species-level variation in CAZyme repertoires within the *Bacteroides* genus, the other genera appeared less heterogeneous, also because they have considerably fewer species members in the human gut (Fig. 3a and Extended Data Fig. 2). While *B. intestinihominis* has been described as a mucin specialist that can grow exclusively on mucin O-type glycans^23^, this has to our knowledge not been reported for the two *Coprobacter* spp., which our results suggest are prolific mucin foragers. Lastly, we found *Hungatella* and *Eisenbergiella* genomes to be strongly enriched in both GAG and mucin-targeting CAZymes (Fig. 2a), with the overall CAZyme repertoire of *H. hathewayi* and *E. tayi* being similar to known mucin foragers (Figs. 2b,c and 3a).

To obtain further evidence for the potential mucin-metabolising capacity of *E. tayi* and *H. hathewayi*, we screened their genomes for colonic mucin-targeting sulfatases (S1_4, S1_11, S1_15, S1_16 and S1_20) using the SulfAtlas tool^24^. This revealed the genomic presence of most known colonic-mucin targeting sulfatases in *E. tayi* and *H. hathewayi* (Supplementary Fig. 2). Taken together, our analysis gives a systematic overview of glycan-related metabolic potential of human gut bacterial genera and species (Fig. 2 and Extended Data Figs. 1 and 2).

## Experimental validation of mucin-utilization potential by *H. hathewayi* and *E. tayi*

**Extended Data Fig. 3:**
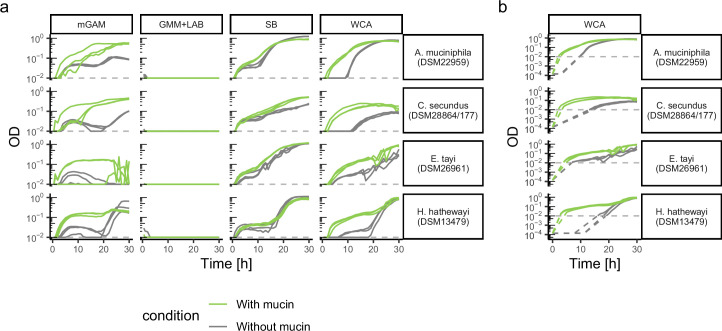
Growth curves of bacterial species on various media with and without mucin. (**a**) Growth curves of *Eisenbergiella tayi* DSM26961, *Hungatella hathewayi* DSM13479, *Coprobacter secundus* DSM28864/177 and *Akkermansia muciniphila* DSM22959 in 3 undefined and 1 defined rich media, with and without mucin (Methods). The y-axis shows the media background-corrected optical density (OD 578 nm) on a log10 scale. Data represent three biological replicates. (**b**) For WCA we observed exponential as well as differential growth between mucin conditions for some strains and hence estimated lag-phase below limit of detection of OD578 < 0.01 based on initial OD of inoculum and the growth rate in exponential phase measured after the cultures passed the limit of detection. The data shown here for *Eisenbergiella tayi* and *Hungatella hathewayi* is identical to the one shown in Fig. 3b. Source data

**Extended Data Fig. 4:**
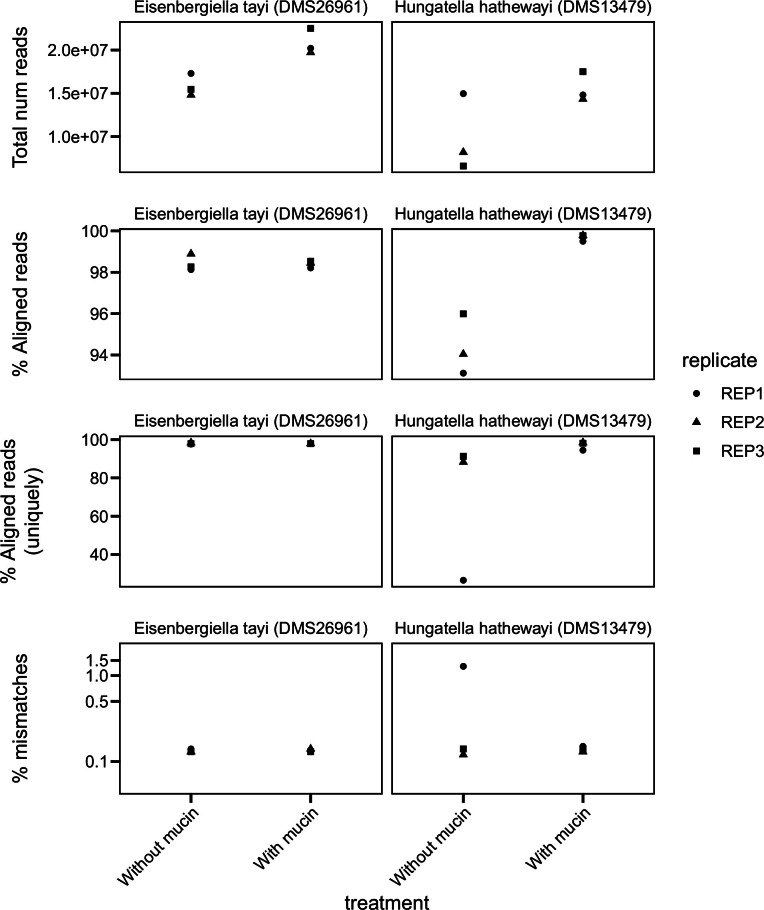
Quality control RNA-Seq data. RNA-seq data quality control metrics. From top to bottom: Total number of generated RNA-seq reads, percent aligned reads against strains’ respective genomes, percent uniquely aligned reads against strains’ respective genomes, percent mismatches (aggregated over all reads) of reds against strains’ respective genomes. Source data

To experimentally test whether *H. hathewayi*, *E. tayi* and *Coprobacter secundus* are able to utilize mucin, we grew these species in media with and without mucin (Extended Data Fig. 3; Methods). We observed better growth (faster outgrowth, increased growth rates and/or higher yields) in the presence of mucin for all three species and the positive control, *Akkermansia* *muciniphila*, in Wilkins–Chalgren Anaerobe (WCA) medium (Fig. 3b and Extended Data Fig. 3). To identify which CAZymes may facilitate mucin metabolization, we assessed the transcriptional activity of *E. tayi* and *H. hathewayi*, the two species that grew to higher yields in WCA, at timepoints where cultures were reaching their plateau (Fig. 3b and Extended Data Fig. 4). After performing RNA sequencing (RNA-seq), we computed differentially abundant genes in the presence and absence of mucin (Methods). This analysis revealed several upregulated CAZymes in both species, pinpointing proteins putatively involved in mucin metabolization (Fig. 3c). These results show that *H. hathewayi* and *E. tayi* grow better in rich media supplemented with mucin, suggesting that they can metabolically utilize mucin, thereby supporting our computational predictions.

## Meta-analysis of HIS versus LMIS gut metagenomes

**Fig. 4:**
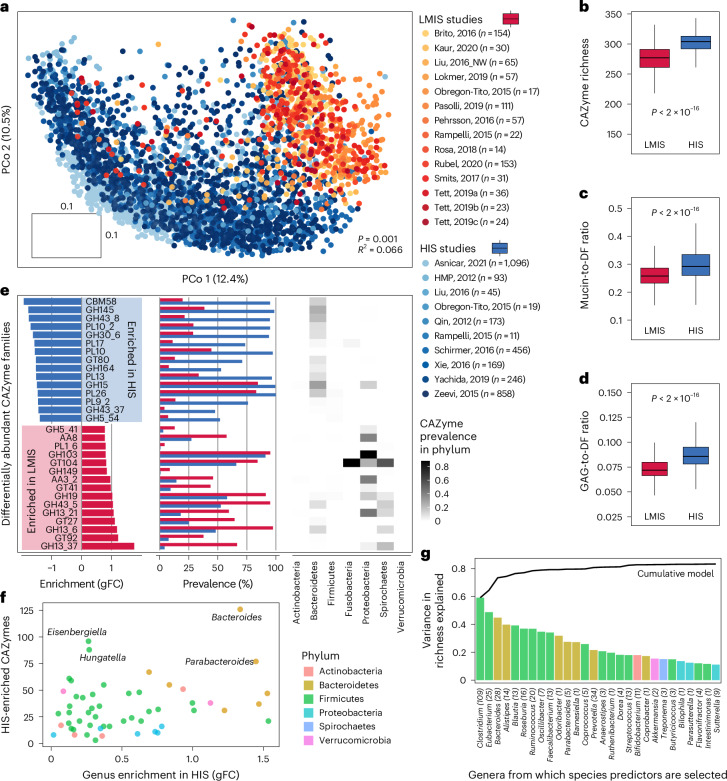
Meta-analysis of CAZymes comparing metagenomes between HIS and LMIS. **a**, Principal coordinate analysis using pairwise Canberra distances between gut microbiome CAZy profiles of HIS and LMIS individuals (*n* = 3,960; Extended Data Fig. 5). PERMANOVA was applied for testing group differences. Data from the references listed in Supplementary Table 4. **b**, Box plots of the number of unique CAZymes between HIS (*n* = 3,166) and LMIS (*n* = 794) individuals. **c**, Box plots of the ratio between the total abundance of mucin-targeting and DF-targeting CAZymes between HIS (*n* = 3,166) and LMIS (*n* = 794) individuals. **d**, Box plots of the ratio between the total abundance of GAG-targeting and DF-targeting CAZymes between HIS (*n* = 3,166) and LMIS (*n* = 794) individuals. For the box plots in **b**, **c** and **d**, the centre value corresponds to the median, the box indicates the interquartile range and whiskers extend to 1.5 times the interquartile range. Significance was tested using unpaired, two-sided Wilcoxon tests. **e**, Bar plots showing the 15 most strongly HIS-enriched (blue) and 15 most strongly LMIS-enriched (red) CAZyme families based on gFC, their prevalence and a heat map of mean phylum-level prevalences of these families based on genomic data. **f**, Genus enrichment in HIS individuals versus the number of HIS-enriched CAZymes encoded per genus (calculated as explained in **e**). **g**, Variance in community CAZyme richness explained by microbial species abundances grouped by genus. Bar plots show the *R*^2^ values of linear models based on mOTU^55^ abundances from within a single genus. Line plot indicates the *R*^2^ value of a cumulative model based on all mOTUs up to a given genus. The numbers in parentheses correspond with the number of mOTUs for the given genus (Methods). Source data

Turning from individual gut microorganisms to community CAZyme repertoires, we studied how these differ across human populations by applying Cayman to publicly available HIS and LMIS gut metagenomes (*n* = 3,166 from HIS and *n* = 794 from LMIS individuals; Supplementary Table 4). Principal coordinate analysis of CAZy profiles showed clear separation between HIS and LMIS microbiomes (permutational multivariate analysis of variance (PERMANOVA), *R*^2^ = 0.066, *P* = 0.001; Fig. 4a).

Previous smaller-scale studies have described that metagenomes from LMIS countries have a higher CAZyme diversity compared with HIS metagenomes, a phenomenon mostly attributed to the low DF content in HIS diets^12–14^. By sharp contrast, we found a consistently higher number of unique CAZymes in HIS compared with LMIS populations, even when only considering CAZymes involved in DF metabolism (Fig. 4b and Extended Data Fig. 6). While we noticed a higher read-mapping rate in HIS compared with LMIS metagenomes (Extended Data Fig. 7), an additional comparison of HIS and LMIS samples with similar read-mapping rates rendered this explanation unlikely (Extended Data Fig. 7).

Next, we leveraged our substrate annotation scheme to compare HIS with LMIS CAZyme repertoires. We confirmed previous findings that the abundance ratio between mucin-targeting and DF-targeting CAZymes is higher in HIS compared with LMIS metagenomes (Fig. 4c). Similarly, HIS metagenomes have a higher abundance ratio of CAZymes targeting GAGs (animal-derived glycans; sources for intestinal GAGs include shed epithelial cells and dietary intake) relative to those targeting DF (Fig. 4d). These results are consistent with HIS diets generally containing high amounts of animal-based products and a low fibre content, in contrast to many LMIS diets being more plant based and fibre rich, although large variation exists among these^25–27^. Lastly, we performed substrate enrichment analysis across more refined levels of our substrate hierarchy, revealing that HIS metagenomes were enriched in CAZymes involved in glycoprotein metabolism and, interestingly, also in various NSPs such as pectins and gums (Extended Data Fig. 6d). Given that pectin and gum are commonly added emulsifiers in processed foods, these enrichments may result from a higher intake of processed foods in HIS populations^28^. Together, HIS metagenomes have an increased richness and abundance of gut microbial metabolic potential for degrading host and animal-derived glycans and for NSPs that are common additives in processed foods.

We then subjected each CAZyme family to differential abundance testing between HIS and LMIS using linear models (Fig. 4e). This analysis indicated many CAZymes to differ significantly in abundance (adjusted *P* < 0.05 for 397/457 CAZyme families) and in prevalence (Fig. 4e). Among the most LMIS-enriched CAZymes, we observed four families (GH13_37, GH13_6, GH13_21 and GH13_42) that target resistant starch, consistent with the generally higher intake of whole grains and legumes in LMIS populations^29^. To attribute differentially abundant families to their likely taxonomic origin, we compared the CAZyme prevalence over different phyla (Methods). This revealed that many LMIS-enriched CAZymes are prevalent in Proteobacteria and Spirochaetes (Fig. 4e). The latter phylum has been labelled a ‘vanish’ taxon, common in LMIS but lost in HIS microbiomes^12^. By contrast, the most HIS-enriched CAZyme, CBM58, is part of the SusG protein, which is crucial for starch utilization in *Bacteroides* species, which tend to occur at higher abundance in HIS individuals (Extended Data Fig. 8).

To assess whether *Bacteroides* expansion could explain the increased CAZyme richness in HIS populations, we asked how many HIS-enriched CAZymes each (HIS-enriched) bacterial genus could contribute to the community repertoire. This analysis identified *Bacteroides* to have the largest number of HIS-enriched CAZymes (*n* = 126; Fig. 4f). We next investigated to which extent the genomic CAZyme repertoire of a small number of key taxa would be predictive of the whole community’s CAZyme richness across human populations. To this end, we constructed linear regression models for CAZyme richness from genus abundances with stepwise increasing sets of taxonomic predictors (Methods; Fig. 4g). CAZyme richness could be predicted well by a few genera (*R*^2^ value of 0.76 with top 5 predictive genera). However, while *Bacteroides* was among these predictors, *Eubacterium* and *Clostridium* were even more predictive of community CAZyme richness. These results indicate that while HIS-enriched *Bacteroides* is genomically the richest in HIS-associated CAZymes, its abundance variation alone does not explain community CAZyme content. Instead, several key taxa together appear to better explain the interindividual differences in CAZyme richness across human populations.

**Fig. 5:**
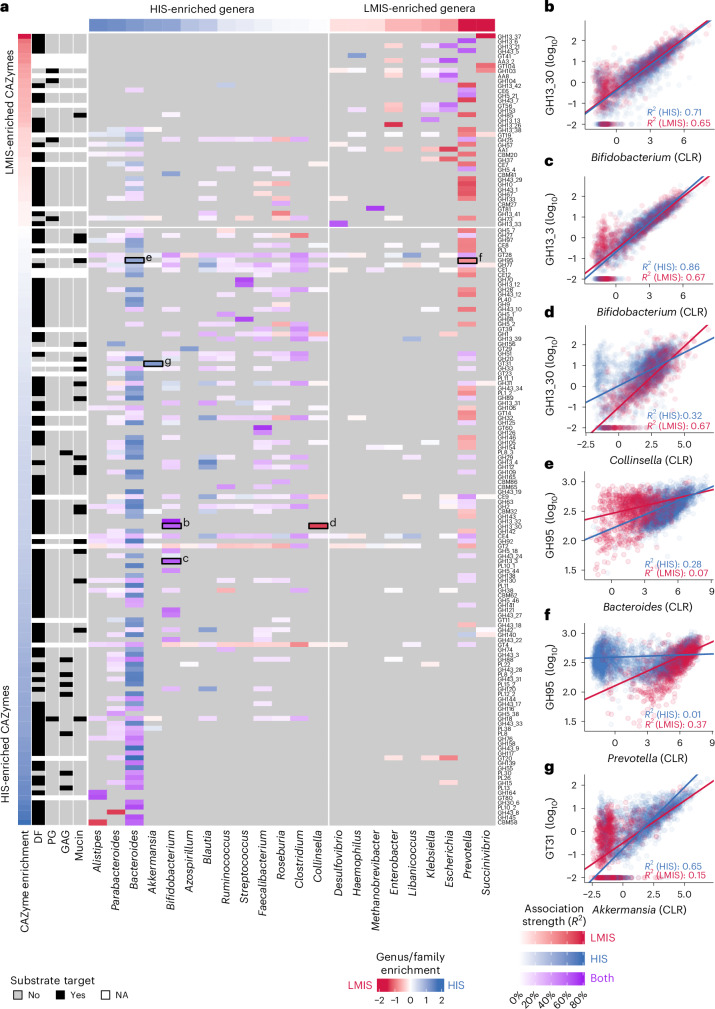
Connecting taxonomic composition to CAZyme repertoires in HIS and LMIS microbiomes. **a**, Heat map showing predicted taxonomic contributions to CAZyme abundances in HIS (*n* = 3,166) and LMIS (*n* = 794) gut microbial communities. Coloured cells depict the *R*^2^ value of multivariable linear models in which log_10_-scaled CAZyme abundance values were predicted from all CLR-transformed species (mOTU) abundances within a given genus. Blue and red cells indicate better fits in HIS or LMIS models, respectively, while purple cells indicate similar fits for HIS and LMIS models (*R*^2^ values within twofold difference). Grey cells indicate models with an insignificant fit after multiple testing correction at 1% FDR. HIS versus LMIS enrichments for taxa and CAZy families were defined as previously (Fig. 4) and substrate annotations for CAZymes are included as the left-most columns. **b**–**g**, Scatter plots between taxonomic abundances of different genera and abundances of different CAZymes: between *Bifidobacterium* abundance and GH13_30 family abundance (**b**), between *Bifidobacterium* abundance and GH13_3 family abundance (**c**), between *Collinsella* abundance and GH13_30 family abundance (**d**), between *Bacteroides* abundance and *GH95* family abundance (**e**), between *Prevotella* abundance and GH95 family abundance (**f**), and between *Akkermansia* abundance and GT31 family abundance (**g**). In all of these cases, genus- and mOTU-level associations with CAZyme abundance were similar (Extended Data Fig. 9). Linear fits are indicated by *R*^2^ values (Methods). Source data

To connect taxonomic composition to community CAZyme repertoire, we built linear regression models to predict the abundance of each CAZyme family from species abundance profiles within each gut microbial genus. We trained these models separately for HIS and LMIS populations to compare the associations (Methods). We found strong pairwise associations between taxonomic and CAZyme abundances and, as expected, we observed the contributions to CAZyme abundances of highly abundant genera, such as *Bacteroides* and *Prevotella*, to generally outweigh those of rare, low-abundant genera (Fig. 5a). However, we noted exceptions to this trend, for example, *Bifidobacterium* with GH13_30 and GH13_3 (Fig. 5b,c). GH13_30 is well characterized in *Bifidobacterium* species^17^ and therefore the strong association with *Bifidobacterium* abundance appears plausible. By contrast, GH13_3 has not been experimentally characterized in any gut microorganism^17^, yet our analysis shows GH13_3 abundance to be very well predictable from *Bifidobacterium* spp. abundances, suggesting this genus to be the sole contributor to the GH13_3 family in both HIS and LMIS human gut microbiomes. Second, we observed that some associations differ between HIS and LMIS individuals despite taxa being similarly abundant: For example, *Collinsella* is strongly predictive of GH13_30 abundance in LMIS individuals but less so in HIS individuals (Fig. 5d). The fact that *Collinsella* does not differ strongly in abundance suggests that other taxa contribute to this CAZyme pool in HIS but not LMIS individuals. An ecologically distinct example is GH95 (encoding an α-fucosidase): While in HIS individuals this CAZyme family strongly associates with *Bacteroides* (Fig. 5e), but not with *Prevotella* (Fig. 5f), the opposite pattern was observed for LMIS individuals. This suggests that in HIS, *Bacteroides* has taken over functionalities originally provided by *Prevotella*. This example is especially interesting given that *Prevotella* is generally not regarded as a mucin forager. In addition, no enzyme from *Prevotella* has been experimentally identified as GH95, even though this family has been characterized in numerous gut microorganisms^17^. As a final example, *Akkermansia* is highly predictive of GT31 in HIS but not in LMIS individuals (Fig. 5g), indicating that *Akkermansia* is the principal carrier of this CAZyme family in HIS individuals but not in LMIS individuals. By contrast, in LMIS individuals, the single-celled parasite *Blastocystis* (whose genome also encodes GT31) is more strongly associated with GT31 abundance than *Akkermansia* (Supplementary Fig. 3).

## Meta-analysis of CRC case–control studies

HIS lifestyle is associated with many common diseases, including CRC^30^. Differences in gut microbial composition between patients with CRC and controls are well understood^31^ and differences in their CAZyme repertoire have also been reported in individual studies^8^. Here, we provide a detailed assessment of gut CAZyme repertoires by applying Cayman in a meta-analysis of 1,998 metagenomes (*n* = 968 patients with CRC and *n* = 1,030 controls; Supplementary Table 5) from four continents and ten different countries.

**Fig. 6:**
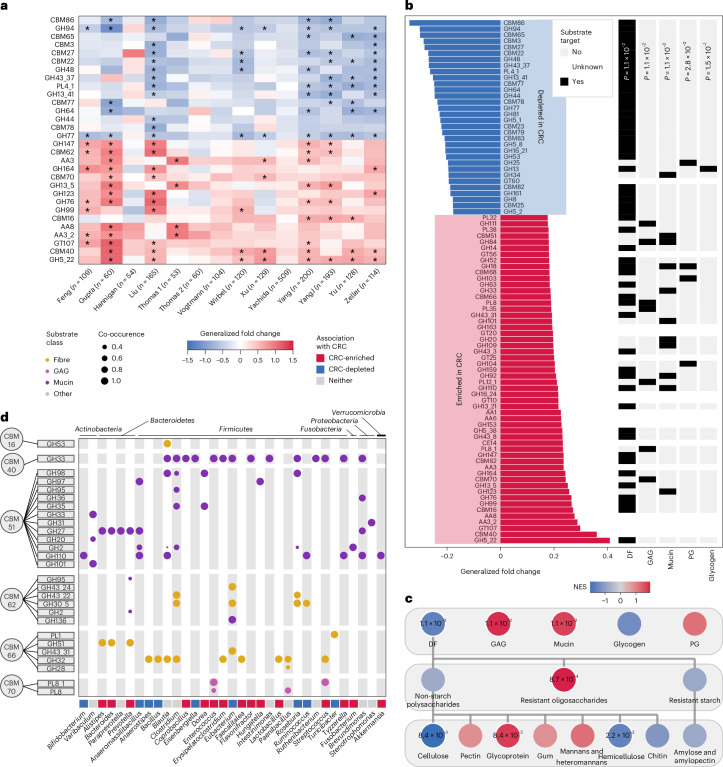
Meta-analysis of CAZymes in metagenomes from patients with CRC and tumour-free controls. **a**, Heat map with univariate associations per CRC study. Asterisk signifies per-study FDR-corrected significance (BH-adjusted *P* value <0.05). The top and bottom 15 families were selected on the basis of the order of CAZyme families from the meta-analysis in **b** (all having a BH-adjusted *P* value <0.05). *P* values for differential CAZyme abundances were calculated using linear models with disease status as predictor. Data from the references listed in Supplementary Table 5. **b**, Meta-analysis of CAZyme enrichments over all studies using LMMs where CAZyme abundances were predicted from disease status as in **a** but additionally with study as a random effect. Subsequently, all CAZyme families were subjected to GSEA to identify overrepresented substrate groups. **c**, GSEA analysis at different hierarchical substrate levels to identify overrepresented substrate groups differentially targeted between CRC and control metagenomes. NES, normalized enrichment score. **d**, Dot plot depicting CRC-enriched CBMs co-occurring with catabolic CAZyme families within the same gene. Co-occurrences were calculated using a modified Jaccard index on bacterial genomic data (Methods). Dot size corresponds to the strength of association, and colour indicates substrate annotation of the enzymatic domain. Source data

We first computed differentially abundant CAZyme families per study (Fig. 6a; Methods), revealing broadly consistent CAZyme enrichments and depletions in patients with CRC across studies (Fig. 6a); however, in line with a previous meta-analysis, several datasets did not show significant differences when assessed in isolation^31^.

To perform a meta-analysis of differentially abundant CAZyme families while accounting for study heterogeneity, we fitted linear mixed models predicting cancer state from CAZyme profiles with study as a random effect. Out of 459 CAZymes, 282 were significant at a 5% false discovery rate (FDR) (Fig. 6b). We next aimed to identify potential community-wide shifts in carbohydrate substrate preferences by leveraging our substrate scheme in a gene set enrichment analysis (GSEA; Methods). We found that CRC metagenomes were significantly depleted in CAZyme families with DF-derived substrates (*P* = 1.1 × 10^−2^), while we also noted an enrichment in GAG and mucin substrates (Fig. 6b; *P* = 1.1 × 10^−2^ and *P* = 1.1 × 10^−2^, respectively). Mucin-to-DF and GAG-to-DF ratios further confirmed this finding, with both being increased in CRC metagenomes (Extended Data Fig. 10). Substrate enrichment analysis across different levels of granularity revealed a significant depletion of cellulose and hemicellulose substrates (*P* = 8.4 × 10^−3^ and *P* = 2.2 × 10^−2^, respectively), among others (Fig. 6c). Taken together, these results showed a clear enrichment of host and animal glycan substrates in CRC metagenomes, while DF-derived substrates were depleted. These findings align with earlier reports^8,31^ and dietary epidemiological risk factors for CRC^32–34^ and might be a consequence of dietary differences or ecological adaptations of the gut microbiome to increased availability of host glycans in CRC.

To learn more about the substrates of CRC-enriched CBMs, we investigated whether CBMs co-occurring with enzymatic domains (GHs or PLs) on the same gene^35^ could predict substrate binding preferences. This co-occurrence analysis across our bacterial genome collection (Methods) identified several consistently co-occurring pairs (Fig. 6d), some of which were taxonomically conserved across phyla (for example, CBM40-GH33 and CBM51-GH110), while others were more restricted (for example, CBM16-GH53 specific to *Blautia* and CBM51-GH101 specific to *Varibaculum*). Comparing the substrate annotations of co-localized CBM and GH/PL domains, we confirmed several previously reported associations: For example, the consistent and specific co-occurrence of CBM40 and GH33 across multiple gut bacterial genera indicated that CBM40, and not only GH33, facilitate mucin foraging^36,37^. Similarly, CBM70 specifically co-occurred with PL8 (and its subfamily PL8_1), a known GAG-degrading enzyme, consistent with a demonstration of CBM70 binding the GAG hyaluronan^38^. It should be noted that while CBMs and enzymatic domains often target the same substrate, this is not always the case, as was shown for, for example, xylanases in combination with cellulose-binding CBMs^39^. Similarly, while CBM62 is known to co-occur with DF-targeting GH families (Fig. 6d), we additionally found it co-occurring with the mucin-targeting GH136, which predicts CBM62 to perhaps have broader glycan-binding specificity. Taken together, this co-occurrence analysis revealed interesting conservation of CAZyme domain pairs and suggests that substrate specificity of CAZyme domains could be predicted more broadly using a ‘guilt-by-association’ approach^40^.

## Discussion

Here, we present Cayman, an open-source software tool for profiling CAZymes from metagenomic data, along with a hierarchical substrate annotation for CAZymes. Together, this enables easy and scalable functional analysis and interpretation of the CAZyme repertoires of microbial communities. Our CAZyme analyses of the human gut microbiome extend beyond an important previous study inferring gut microbial CAZyme repertoires on the basis of an artificial ‘mini-microbiome’ (constructed from 177 genomes)^2^, which is unlikely to generalize to in vivo microbiomes owing to biased representation and unaccounted abundance differences. However, our analysis also shares limitations with this and other studies: CAZyme and substrate annotations are transferred via sequence similarity. All such approaches tend to miss functional consequences (for example, substrate changes) of subtle differences in protein sequence and are unaware of context, that is, function is ascribed considering each gene in isolation. These limitations make it challenging to infer functions that require multiple enzymes, such as cellulose degradation, for which it remains unclear if performed by HIS gut microbiota at all^41^. A related challenge is the precise assessment of strain-specific functions, as has, for example, been shown for the important gut bacterium *Bacteroides ovatus*^23^.

Nevertheless, Cayman enables surveys of gut microbial CAZyme repertoires across human gut microbial (meta-)genomic datasets. Our applications particularly suggested *E. tayi* and *H. hathewayi* to be capable of mucin utilization, while they were not previously appreciated as such. While experimental data on mucin utilization for *E. tayi* had been lacking, its abundance had been observed to increase in communities after supplementing mucin to culture media and bioreactors^42,43^. For *H. hathewayi*, GAG-metabolising capacity was previously shown^44^, but regarding potential mucin-metabolising capacity, *H. hathewayi* had only been observed to increase in rodent guts in response to dietary supplementation of porcine gastric mucin^45^. Here, we provided direct evidence, through growth experiments and differential gene expression analysis, that *E. tayi* and *H. hathewayi* metabolize mucin, which confirms our computational predictions. However, it should be noted that our experiments used (impure) gastric porcine mucin^46^. To definitively establish these bacteria as human gut mucin foragers, showing growth on a minimal medium with purified human colonic mucin as the sole carbon source would be desirable.

Our comparison between HIS and LMIS gut microbiomes revealed the unexpected observation of higher CAZyme richness in HIS individuals, which seemingly contradicts current consensus^12,14,19^. There are multiple possible explanations for this discrepancy that support our conclusion: First, we used a larger and more diverse collection of both HIS and LMIS datasets than previous studies. Previous work undersampled HIS populations in particular: for instance, Smits et al. compared exclusively against the data from the first phase of the Human Microbiome Project^9^, which exhibits an unusually low number of unique CAZymes (consistent with previously observed low taxonomic diversity^47^). Second, utilization of a gene catalogue in Cayman as compared with direct CAZyme annotation of reads^14^ or assembled contigs^19^ is expected to yield more accurate results because complete genes and not just short reads are annotated competitively (reducing spurious matches)^21^. Biologically, increased CAZyme richness in HIS individuals is not implausible given the year-round availability of a wide variety of foods (containing diverse types of fibres) in contrast to food sources in traditional LMIS populations being more restricted by seasonality and geographic proximity^14,48^. To better understand how diet and other lifestyle factors shape gut microbiome functions in general and CAZyme repertoires in particular, individual-specific dietary intake and gut metagenomic data will be instrumental, ideally also tracking variation over time. If combined with systematic in vitro growth data assessing the effects of various dietary components on specific gut bacteria, a much more highly resolved analysis of the glycan utilization capabilities of microbial communities could be performed.

In conclusion, Cayman was instrumental for gaining insights into CAZyme biology of the human gut microbiome and we anticipate it to be broadly useful for the study of other microbial communities.

## Methods

### Generation of CAZyme (sub)family module sequence set

To obtain family-wise CAZy modules, we first downloaded CAZy sequences^49^ (HMMdb release 9.0 and CAZyDB released on 30 July 2020) from a total of 676 families and subfamilies. We then extracted CAZy modules from these sequences using the dbCAN pHMMs (from the same dbCAN2 release) using an *E*-value threshold of 1 × 10^−15^ and a coverage threshold of 0.35. Next, we generated family-wise multiple sequence alignments of module sequences using mafft-linsi^50^ (v7.505) on representative module sequences obtained from mmseqs2 (release 15-6f452) with–easy-cluster–min-seq-id 0.99–cov-mode 0 -c 0.5^51^. Default parameters were used unless stated otherwise.

### Optimization of pHMM *P* value cut-offs

We set out to determine family-specific sequence similarity cut-offs for pHMMs for optimized detection of CAZymes in large sequencing datasets. To this end, we constructed and evaluated pHMMs for each CAZy family individually using blocked cross-validation: For each CAZy family and cross-validation fold, we divided family sequences into training (~80%) and testing sets (~20%): The training set was used to build the family pHMM and the test set was used as positive instances at testing time, while sequences from other CAZyme modules were used as negative instances (for more details, see below). When building pHMMs from training sequences, we omitted sequences shorter than 80% of the median module length. For testing, we used entire gene sequences (instead of family module sequences for training pHMMs) because they represent a more realistic search space. Training and test fold generation was done in a blocked fashion, where similar sequences remain together in either the training or test set. This was done to minimize information leakage from the training set into the test set. To define blocking groups, module sequences within each family were clustered at 60% sequence identity using mmseqs2 (arguments easy-cluster, --min-seq-id 0.60, --cov-mode 0, -c 0.5). Folds were designed in such a way that test sets never overlap with each other.

We evaluated families differently depending on their hierarchy of subfamilies: When evaluating a family without subfamilies, we considered corresponding family sequences as positive instances and all other sequences as negative instances. When evaluating a family with subfamilies, we proceeded as above but additionally considered all subfamily sequences under that family as positive instances. When evaluating subfamilies, we ignored upstream family sequences and considered all other families (including sister subfamilies) as negative instances. In all cases, we furthermore utilized non-CAZy sequences from UniProt as additional negative instances. These sequences were obtained in the following manner. First, all manually curated sequences (Swiss-Prot) with an annotation score of five out of five (indicating experimental evidence at protein level) were downloaded (*n* = 54,978 sequences, 5 March 2021). We subsequently filtered out all sequences with an annotated Enzyme Commission number present in CAZy, yielding 51,507 sequences. As CBMs are non-catalytic (and thus have no Enzyme Commission number), we did not add UniProt sequences as negative instances when we evaluated pHMMs for CBMs.

This setup was applied to the 527 CAZyme families that had at least 50 sequences in the CAZy database. For the 130 families that had fewer than 50 sequences, but 5 or more sequences or where fewer than five blocking groups existed, cross-validation was performed without blocking. A total of 11 families had fewer than five sequences and were not cross-validated in this manner but instead were used with the median optimized *P* value cut-off of the corresponding CAZyme class. In this way, we derived cut-offs for a total of 668 CAZy families.

Finally, we determined family- and fold-wise optimal *P* values by iterating over *P* value thresholds and choosing the *P* value that maximizes the F1 score. The F1 score was calculated using the follow formula: 2 × (recall × precision)/(recall + precision). Recall was calculated using the formula: TP/(TP + FN) and precision using TP/(TP + FP), where TP is the number of true positives, FN is the number of false negatives and FP is the number of false positives. We optimized *P* value cut-offs in this manner for 647 CAZy families, and the 21 CAZy families with an F1 score <0.5 were removed, as these CAZyme families could not be reliably detected using HMMs.

### Annotating gene sequences

To annotate genomic sequences, we used PyHMMER^52^ (version 0.10.15) to run all rebuilt pHMMs against the genomic sequence set and filtered the hits using the family-wise optimized *P* value cut-offs (see above). Next, we kept only those residues where at least half the fold-specific HMMs (rounded up) yielded a significant hit. Finally, we merged overlapping regions to obtain the annotations as simple coordinates on a genomic sequence.

### Substrate annotation scheme design

To design a meaningful, hierarchical substrate scheme, we aimed to capture for an extensive list of glycans, the origin of the glycan, its function in the organism from which it originated (storage or structural glycan) and its function at destination (especially relevant for mammalian gut environments), which was further subdivided into three different categories. The three subcategories of function at destination were based on recommendations^20^ that divide DF into several subclasses (for example, whether a glycan derives from an NSP or a resistant starch and in which category the class of poly- or oligosaccharide falls). We then applied the same logic to glycans that are not DFs, such as GAGs and mucin-associated glycans. After having constructed a complete table for selected glycans (Supplementary Table 2) with a hierarchical substrate design, we continued to manually curate all CAZyme (sub)families in the CAZy database. This annotation effort led to glycan-specific annotations of each individual CAZyme (sub)family. By subsequently mapping our Supplementary Table 2 onto all individual CAZy (sub)families, we then obtained a large table (Supplementary Table 1) that, for every individual CAZyme family, contained information on the hierarchical categories we designed in Supplementary Table 2. All substrate annotations were initially performed by Q.R.D. and H.L.P.T. and were subsequently independently validated by O.D.-B.

### Genomic annotation of human gut microbial CAZymes

Genomic analysis was based on all high-quality genomes (completeness >90%, contamination <5%) from Almeida et al.^15^, amounting to a total of 6,456 isolate genomes and 101,229 MAGs, which we annotated with our pipeline. The taxonomic tree in Fig. 2 shows 91 prevalent human gut bacterial genera, defined as follows: On the basis of HIS and LMIS control samples (see ‘HIS and LMIS datasets’ section; Supplementary Table 4), we first computed metagenomic operational taxonomic units (mOTUs) that are more than 5% prevalent and whose maximum relative abundance exceeds 1% in at least one sample. We defined a CAZyme family to be present in a genus or species if at least 20% of genomes within that taxon carried at least one copy of the family.

### Preprocessing of metagenomic datasets

Before functional profiling, raw reads were subjected to the following protocol with bbduk (bbmap-version 38.93): (1) low-quality trimming on either side (qtrim = rl, trimq = 3), (2) discarding of low-quality reads (maq = 25), (3) adaptor removal (ktrim = r, *k* = 23, mink = 11, hdist = 1, tpe = true, tbo = true; against the bbduk default adaptor library) and (4) length filtering (ml = 45). The cleaned reads were screened for host contamination using kraken2 (version 2.1.2)^53^ against the human hg38 reference genome with ribosomal sequences masked (SILVA 138).

### Cayman profiling steps for obtaining CAZyme abundances from cleaned metagenomic shotgun sequences

As input to Cayman, cleaned metagenomic shotgun reads are mapped to the habitat-specific Global Microbial Gene Catalogue^21^ (which we further filtered for genes with a prevalence >0.5% to improve memory footprint and runtime required for profiling) using BWA-MEM (0.7.17) with default parameters and name sorted by samtools collate (1.14)^54^. Alignments are then filtered to >45 bp alignment length and >97% sequence identity, which results in very high mapping rates (Extended Data Fig. 6). Cayman quantifies CAZyme domain abundances by counting reads overlapping annotated CAZy domains. Paired-end reads contribute 0.5 counts per mate, and reads from single-end libraries contribute 1 count. If reads align to multiple domains, they fractionally contribute towards each domain. To account for biases introduced by gene length and sequencing depth, read counts were normalized to reads per kilobase per million mapped reads (RPKM) against the number of reads passing the above alignment filters.

### Taxonomic profiling of metagenomic shotgun data

To obtain taxonomic profiles, we used mOTUs with default settings (v3.1)^55^. Preprocessing was done as described in the ‘Preprocessing of metagenomic datasets’ section. *Blastocystis* profiling was done using the same mOTUs approach with eukaryotic marker genes, an overview of which can be found in Supplementary Table 6.

### HIS and LMIS datasets

To investigate differences in CAZyme repertoire between HIS and LMIS populations, we profiled 19 different datasets from 19 different countries and retrieved the definition of Western and non-Western and associated metadata from the curatedMetagenomicData (CMD) package (v3.6.2)^16^. Binary classification of Western versus non-Western was obtained from this resource, and classification is based on the adoption of a westernized lifestyle encompassing different characteristics, as was previously explained in detail^56^. However, because this binary classification into the terms Western and non-Western can be viewed as eurocentric, we here replaced these terms with HIS and LMIS. We exclusively selected samples from healthy controls aged 18 years or older (age categories ‘adult’ and ‘senior’ in CMD) (*n* = 3,960) also if these were infected with a soil-transmitted helminth (*n* = 91/3,960) given the high prevalence of these in many countries in healthy individuals. In cases where individuals were sampled repeatedly, we only retained a single sample (see Supplementary Table 4 for details per dataset).

### Statistical analysis of HIS and LMIS data

CAZyme richness was calculated by counting the number of uniquely observed CAZyme (sub)families within each sample, provided that the abundance was >1 RPKM. Principal coordinates analysis was performed using Canberra distances on the basis of the CAZy RPKM values. Substrate ratios were calculated by summing the RPKMs of all CAZyme families annotated by the given substrate (for example, DF or GAG) and then computing the ratio between the respective values. CAZyme differential abundance analysis was performed using a linear model implemented in SIAMCAT (v2.5.1)^57^, and obtained *P* values were adjusted using the Benjamini–Hochberg (BH) method, with adjusted *P* values <0.05 considered significant. Features were filtered on the basis of having a prevalence of at least 1% in the entire dataset before performing differential abundance analysis. A pseudocount of 0.01 was added to RPKM values before statistical analysis. Generalized fold changes (gFCs) were calculated as implemented in SIAMCAT^57^. Considering that almost all studies only had HIS or only LMIS samples, we could not block by study.

To study the contribution of microbial taxa to community-level CAZyme abundances in metagenomes, we used the lm function in R to predict log_10_-scaled CAZyme RPKM values (with a pseudocount of 0.01) on the basis of centred log-ratio (CLR)-scaled species-level microbial profiles of the LMIS/HIS dataset collection. For each genus–CAZyme pair, we fit two linear models: one for all LMIS samples and one for all HIS samples. For each genus, all mOTUs (that is, species-level taxonomic groups) of that genus were used to predict CAZyme abundances. All associations computed this way were FDR corrected using the BH method at 1% FDR and further filtered for those where both models have a coefficient >0. In the heat map, we only show those genera with at least one association with *R*^2^ > 0.4 and those CAZymes with at least one association with *R*^2^ > 0.4. We finally filtered out all associations where the CAZyme family is genomically absent in the associated genus.

### CRC datasets and statistical analysis

For the meta-analysis of CRC studies (Supplementary Table 5; our re-analysis included all samples from the listed studies), we determined differentially abundant CAZymes between patient and control metagenomes by fitting LMMs with study as a random effect using the SIAMCAT package^57^. Features with less than 1% prevalence were removed before statistical testing. Next, to investigate whether the obtained signatures were consistent across studies, within-study linear models were applied where exclusively the study label (CRC or control) was included as a variable. gFCs were calculated as implemented in the SIAMCAT package, and *P* values were adjusted using FDR correction (BH method), with adjusted *P* values <0.05 considered significant. Substrate ratios were calculated in a linear mixed model setting with the study being included as a random effect. Study-specific ratios are shown in Extended Data Fig. 10. To compute genera differentially abundant in CRC, we used unpaired, two-sided Wilcoxon tests and FDR-adjusted *P* values at 10% using the BH method. Given that all studies were case–control studies, no repeated sampling of individuals was performed.

### GSEA

To investigate whether there are substrate preferences among the CAZymes enriched or depleted in CRC metagenomes, we performed GSEA using the R package fgsea (v1.22.0) and the fgseaMultilevel function with default parameters. As input measure for fold change, gFCs as obtained from SIAMCAT were used. Adjusted *P* values were calculated to determine significance for each substrate.

### Co-occurrence analysis for CRC-enriched CBMs

To compute CAZyme families co-occurring within genes, we computed a modified Jaccard index between all pairs of CAZymes from our CAZy annotations of a large human gut microbial genome collection (see ‘Genomic annotation of human gut microbial CAZymes’ section). Specifically, we first selected all open reading frames (ORFs) that have at least two distinct CAZyme families, after which we computed for all pairs of CAZyme families the numerator of the index as the number of ORFs that contain both CAZyme families. For normalization of this index, we computed the cardinality for both CAZyme families separately (that is, the number of ORFs that contain that given CAZyme family) and normalized the index by the smaller of both values. We computed this index once for each genus containing at least ten genomes. For the visualization of this data in Fig. 5, we restricted the data to catabolic CAZyme families co-occurring with CRC-enriched CBMs with an index of at least 0.2.

### Bacterial culturing and growth curves

To test whether predicted bacteria can utilize mucin under in vitro conditions, we performed liquid growth assays and RNA-seq in the presence and absence of mucin. The bacterial isolates (*E. tayi* DSM26961, *H. Hathewayi* DSM13479, *C. secundus* DSM28864/177 and *A. muciniphila* DSM22959) were received from the Leibniz Institute DSMZ–German Collection of Microorganisms and Cell Cultures. All bacterial species were cultivated at 37 °C under anaerobic conditions in a vinyl anaerobic chamber (COY) inflated with a gas mix of approximately 2% H_2_, 12% CO_2_ and 86% N_2_.

Frozen stocks were streaked onto mGAM agar and WCA agar plates and incubated for 48 h at 37 °C. Three single colonies from each species were precultured in 1 ml mGAM or WCA broth in 96-deep-well plates (Greiner, 2 ml, 651261). All the strains were grown anaerobically in triplicate, twice, for 24 h to ensure robust growth. Optical density (OD) at 578 nm was measured for all species using U-bottom shallow 96-well plates (Fisher Scientific; 168136) containing 100 μl culture with a microplate spectrophotometer (EON, Biotek). We reconstituted all strains to an OD of 0.2 in PBS before starting the experiment. mGAM-diluted OD-0.2 cultures were transferred into different media (see below) with mucin (III) (Sigma; M1778) or without mucin (*n* = 3); whereas WCA-diluted OD-0.2 cultures were transferred only into WCA medium under the same conditions. Specifically, 50 μl of OD-0.2 culture was diluted in 950 μl PBS in a 96-deep-well plate, and, then, 10 μl of this dilution was added to 1,490 μl of respective media 96-deep-well plates, resulting in a final OD of 0.00007 and a total volume of 1,500 μl per well. For each of the species, three samples were prepared with mucin and three without. Three sets of 1.5-ml cultures were grown in deep-well plates and incubated to mid-exponential phase.

Different media used in experiments for cultivation of individual species belong to three undefined media: mGAM (modified Gifu anaerobic medium broth, HyServe; 05433), WCA (Wilkinson–Chalgren anaerobic broth, Oxoid; CM0643) and Schaedler broth (Carl Roth; 5772.1). We used one defined media established previously to grow different human gut bacteria dGMM + LAB (M3)^58^.

To monitor growth, 100 μl of each culture was transferred to a U-bottom shallow 96-well plate and sealed with a breathable membrane (AeraSeal, Sigma). Growth was assessed using a microplate spectrophotometer (EON, BioTek) with OD_578_ (optical density measured at a wavelength of 578 nm) measured every hour for 30 h and orbital shaking for 30 s before each reading. For transcriptomics, cultures in WCA broth were analysed. From WCA-grown cultures, the remaining 1.4 ml was collected at mid-exponential phase (18–22 h), and cells were pelleted at 4,000 rpm for 10 min at 4 °C. Supernatants were removed using a liquid handling robot (Biomek i5, Beckman Coulter), and pellets were stored at −80 °C.

### RNA extraction and sequencing

Total RNA was extracted using the Zymo kit (Direct-zol RNA Miniprep Plus; R2072) following DNase I treatment as per the manufacturer’s protocol. RNA was eluted in ultrapure water and stored at −80 °C. RNA concentration was quantified using a Qubit RNA Broad Range kit (ThermoFisher), and RNA integrity (average RNA integrity no. of 9) was assessed using RNA Nano Chip on a Bioanalyzer (Agilent Technologies) at Genomics Core Facility (EMBL).

After the quality control rRNA depletion, library preparation and sequencing were subsequently performed at Genomics Core Facility (EMBL). rRNA was depleted using the NEBNext Bacteria rRNA Depletion Kit (New England Biolabs). Libraries were prepared on the automated liquid handling system Beckman i7 (Beckman Coulter) using the NEBNext Ultra II Directional RNA Library Prep Kit (New England Biolabs) and sequenced on an Illumina NEXTSeq2000 P2 kit (~450 million reads, 100 bp, single-end). Sample fragmentation time was 9 min and 16 PCR cycles were used.

### RNA-seq analysis

To quality control the RNA-seq data, we visualized total sequencing depth, percentage of (uniquely) aligned reads as well as the mean percentage mismatches of aligned reads. This quality control step suggested issues with one replicate of *H. hathewayi*, which we removed in the downstream analysis (Extended Data Fig. 4). For transcript quantification, we used the nfcore rnaseq workflow (v.3.3.1), which uses Trim Galore! (v0.6.10) for adaptor and quality trimming, STAR (v2.6.1 d) for read alignment against the genome and Salmon (v.1.10.3). We then used DESeq2 (v1.34.0) to compute differentially abundant genes.

### Reporting summary

Further information on research design is available in the Nature Portfolio Reporting Summary linked to this article.

## Supplementary information

Supplementary Information Supplementary Figs. 1–3.

Reporting Summary

Peer Review File

Supplementary Tables 1–6 Excel file containing Supplementary Tables 1–6.

## Source data

Source Data Fig. 2 CAZy genome annotation statistics.

Source Data Fig. 3 Mucin-targeting CAZyme summaries and exponential growth and RNA-seq data statistics.

Source Data Fig. 4 CAZy repertoire statistics comparing gut metagenomes from HIS versus LMIS countries.

Source Data Fig. 5 CAZy repertoire statistics comparing HIS and LMIS countries resolved by families.

Source Data Fig. 6 CAZy repertoire statistics comparing patients with CRC and control metagenomes.

Source Data Extended Data Fig. 1 Copy numbers of CAZymes in bacterial genera.

Source Data Extended Data Fig. 2 Genomic prevalence of mucin-metabolizing genes in relevant bacterial genera.

Source Data Extended Data Fig. 3 Growth data of bacterial species on various media with and without mucin.

Source Data Extended Data Fig. 4 Quality control metrics of RNA-seq data of bacteria grown with and without mucin.

**Extended Data Fig. 5:**
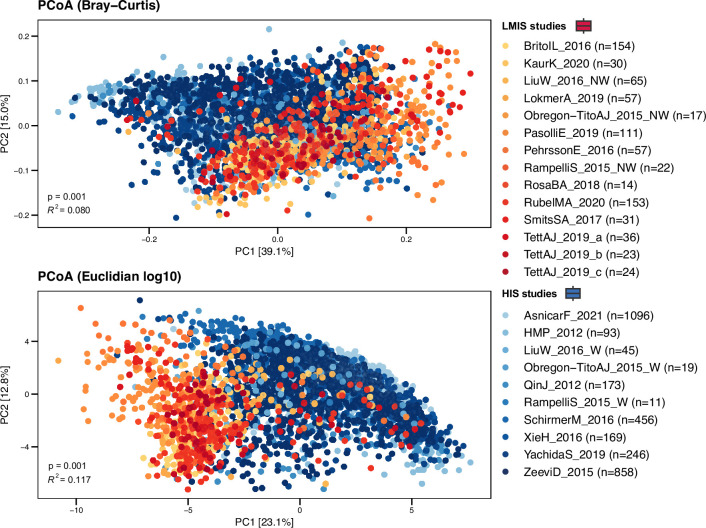
Principal Coordinates Analysis of HIS and LMIS metagenomes. Principal Coordinates Analysis of 3,960 HIS and LMIS metagenomes using Bray-Curtis dissimilarity and log-transformed Euclidean distances. R^2^-values and p-values were computed using PERMANOVA on the pairwise distances between samples. Corresponds to Fig. 3a. Source data

Source Data Extended Data Fig. 5 CAZy repertoire data comparing HIS and LMIS countries resolved by families.

**Extended Data Fig. 6:**
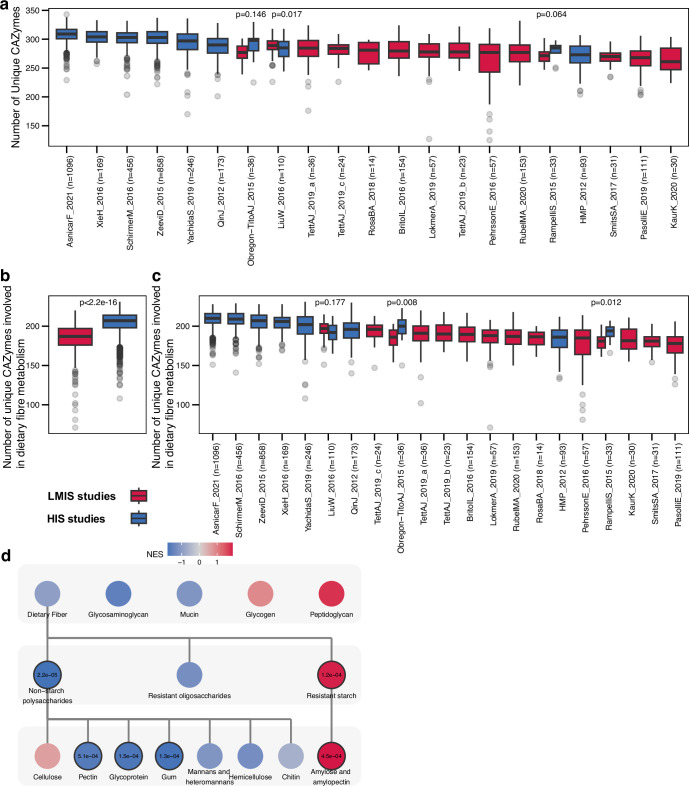
Number of unique CAZymes and enriched substrates across all HIS/LMIS studies. (**a**) CAZyme richness for all studies included in our HIS versus LMIS meta-analysis. Studies are ordered by the median CAZyme richness. P-values were computed using unpaired, two-sided Wilcoxon tests for the studies that had both HIS and LMIS samples included. The indicated (n = ) in the figure refers to the number of samples included in the respective studies (**b**) CAZyme richness of only CAZymes involved in dietary fibre metabolism for the two groups (HIS (n = 3,166) and LMIS (n = 794) individuals) combined. P-value was computed using an unpaired, two-sided Wilcoxon test. (**c**) Same as b, but stratified by all individual studies included in our HIS versus LMIS meta-analysis. Studies are ordered by the median CAZyme richness. P-values were computed using unpaired, two-sided Wilcoxon tests for the studies that had both HIS and LMIS samples included. The boxplots in panels a-c all have a boxplot centre value corresponding to the median, the box indicates the interquartile range, and whiskers extend to 1.5 times the interquartile range. (**d**) GSEA analysis at different hierarchical substrate levels to identify overrepresented substrate groups differentially targeted between HIS and LMIS metagenomes. NES: normalized enrichment score. Source data

Source Data Extended Data Fig. 6 CAZyme richness statistics of all CAZymes, for dietary fibres and summary enrichment statistics of carbohydrate classes in LMIS/HIS individuals.

**Extended Data Fig. 7:**
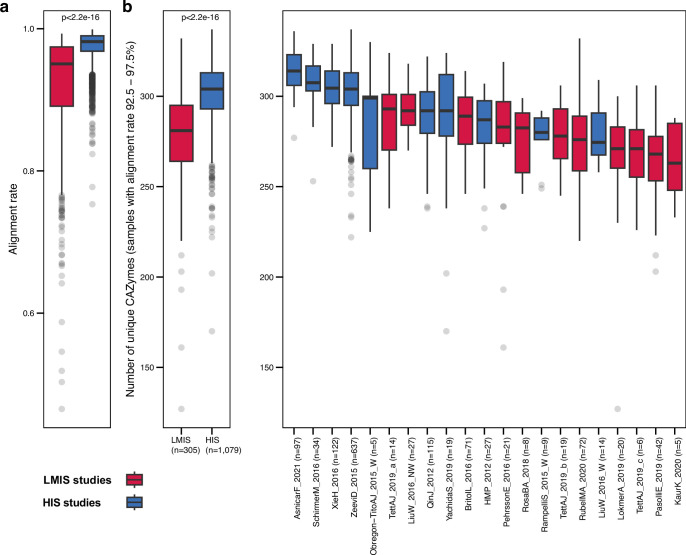
Alignment rates to the reduced GMGC catalogue and number of unique CAZymes on samples with alignment rates between 92.5% and 97.5%. (**a**) Boxplot of metagenome alignment rates against the reduced GMGC catalogue used for CAZyme profiling, contrasting LMIS vs HIS status. P-value was computed using an unpaired, two-sided Wilcoxon test. (**b**) CAZyme richness in HIS/LMIS metagenomes with high alignment rates (92.5% - 97.5%). Studies are ordered by the median CAZyme richness. The indicated (n = ) in the figure refers to the number of samples included in the respective studies with alignment rates 92.5% - 97.5%. P-value was obtained using an unpaired, two-sided Wilcoxon test. All boxplots have a boxplot centre value corresponding to the median, the box indicates the interquartile range, and whiskers extend to 1.5 times the interquartile range. Source data

Source Data Extended Data Fig. 7 Alignment rates data comparing HIS and LMIS countries.

**Extended Data Fig. 8:**
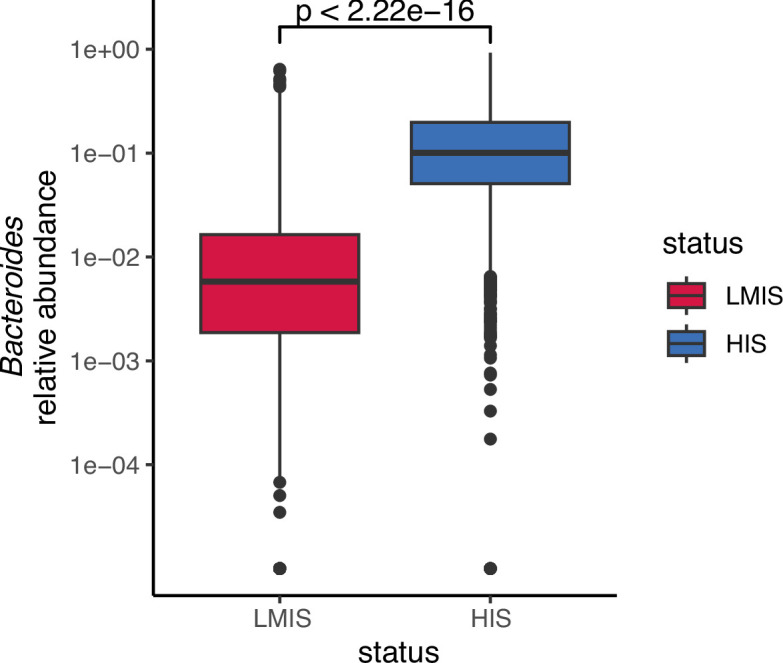
*Bacteroides* relative abundance. Relative abundance of *Bacteroides* in HIS versus LMIS individuals. P-value was obtained using an unpaired, two-sided Wilcoxon test. n = 3,166 for HIS and n = 794 for LMIS. All boxplots have a boxplot centre value corresponding to the median, the box indicates the interquartile range, and whiskers extend to 1.5 times the interquartile range. Source data

Source Data Extended Data Fig. 8 *Bacteroides* relative abundances data comparing HIS and LMIS countries.

**Extended Data Fig. 9:**
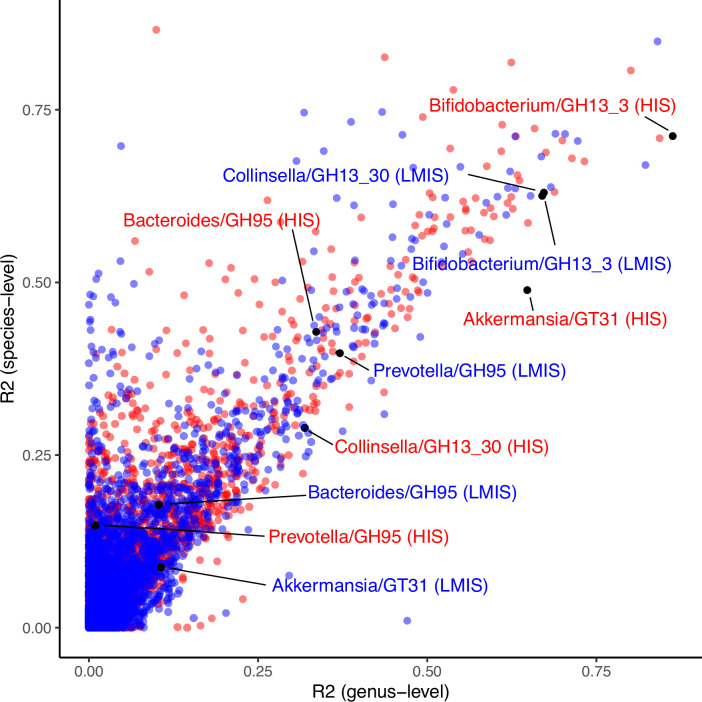
Scatter plot of linear model R2 values predicting CAZyme family abundances from taxonomic profiles. X-axis corresponds to models using genus-level microbial abundance as predictor; Y-axis corresponds to models using mOTU-level microbial abundances as predictor. Label family/genus combinations correspond to examples illustrated in Figs. 4b-4g. Source data

Source Data Extended Data Fig. 9 Associations between bacterial and CAZyme abundances, compared between genus- and species-level aggregations.

**Extended Data Fig. 10:**
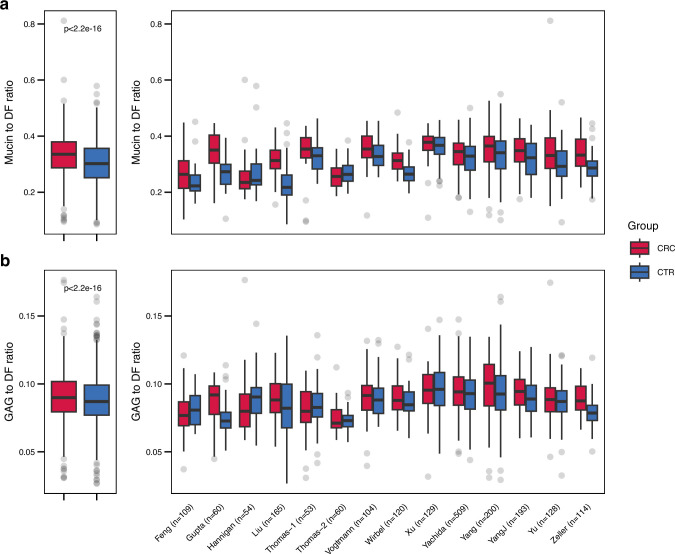
CAZyme Substrate ratios in colorectal cancer patients versus controls. (**a**) Boxplots of the total abundance of mucin-targeting CAZymes normalised by the total abundance of dietary fibre-targeting CAZymes in CRC patients (n = 968) versus controls (n = 1,030). (**b**) Boxplots of the total abundance of GAG-targeting CAZymes normalised by the total abundance of dietary fibre-targeting CAZymes in CRC patients (n = 968) versus controls (n = 1,030). The indicated (n = ) in the figure refers to the number of samples included in the respective studies. P-values for both (**a**) and (**b**) were obtained using linear mixed models with study as random effect. CRC: colorectal cancer, CTR: controls. All boxplots have a boxplot centre value corresponding to the median, the box indicates the interquartile range, and whiskers extend to 1.5 times the interquartile range. Source data

Source Data Extended Data Fig. 10 CAZyme substrate ratio data comparing patients with CRC and control metagenomes.

## Data Availability

All raw metagenomic data used in this study can be accessed from public repositories through the project numbers that are presented in Supplementary Tables 4 and 5. To make our tool as broadly applicable as possible, we annotated all non-human-gut Global Microbial Gene Catalogue (GMGC) sub-catalogues excluding genes with less than 0.5% prevalence^21^ and made the annotations and the gene catalogues available via Zenodo at 10.1101/2024.01.08.574624 (ref. ^59^). CAZyme annotations for all microbial genomes used in this study can also be found in this Zenodo repository. Lastly, RNA-seq data have been deposited under PRJEB90810. Source data are provided with this paper.

## Code availability

Cayman is freely available via GitHub at https://github.com/zellerlab/cayman. All custom code (including required data files) to reproduce the analyses and figures can be found via GitHub at https://github.com/zellerlab/cayman_paper.
