# Supplementary material for: Cayman enables large-scale analysis of gut microbiome carbohydrate-active enzyme repertoires
Source: Nat Microbiol. 2026 Apr 24;11(6):1739–53. doi: 10.1038/s41564-026-02318-2 (PMC13236585; doi:10.1038/s41564-026-02318-2)
Supplement: Supplementary file 1 — Supplementary Figs. 1–3. [file 41564_2026_2318_MOESM1_ESM.pdf]

nature microbiology

Supplementary information https://doi.org/10.1038/s41564-026-02318-2

Cayman enables large-scale analysis of gut microbiome carbohydrate-active enzyme repertoires

In the format provided by the authors and unedited

Supplementary Figure 1

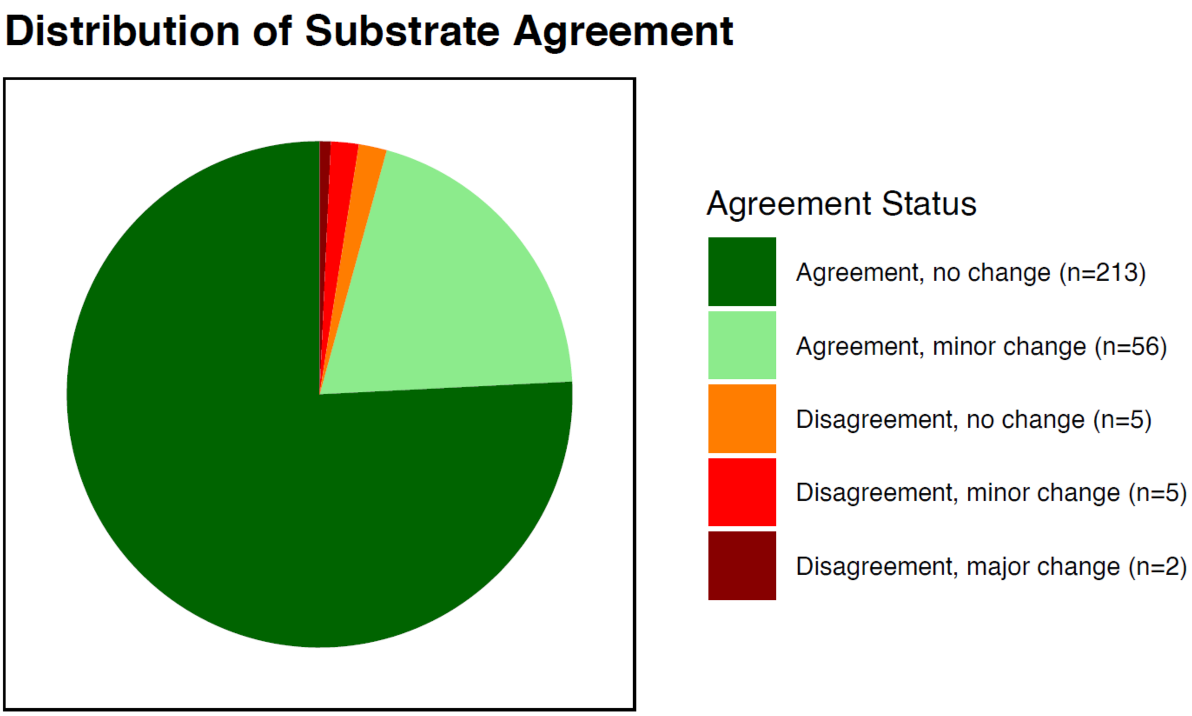

Supplementary Figure 1: Comparison of substrate annotations between dbCAN3 and Cayman for n = 281 CAZyme families that have annotations in both tools.

Supplementary Figure 2

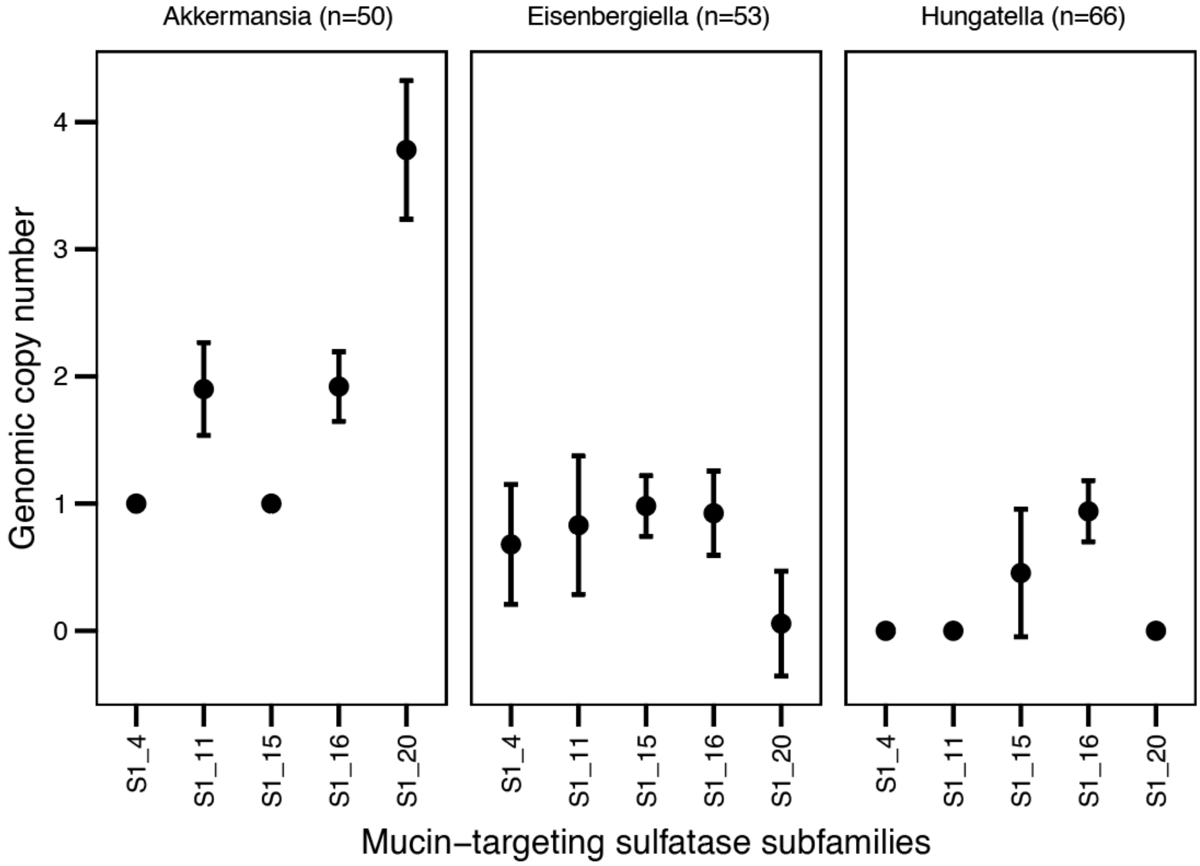

Supplementary Figure 2: Genomic copy numbers of the 5 mucin-targeting sulfatase subfamilies in *Eisenbergiella* spp. (n=53), *Hungatella* spp. (n=66) and *Akkermansia* (n=50) genomes. Error bars indicate 1 standard deviation (upper whisker corresponding to mean + 1 standard deviation and bottom whisker corresponding to mean - 1 standard deviation).

Supplementary Figure 3

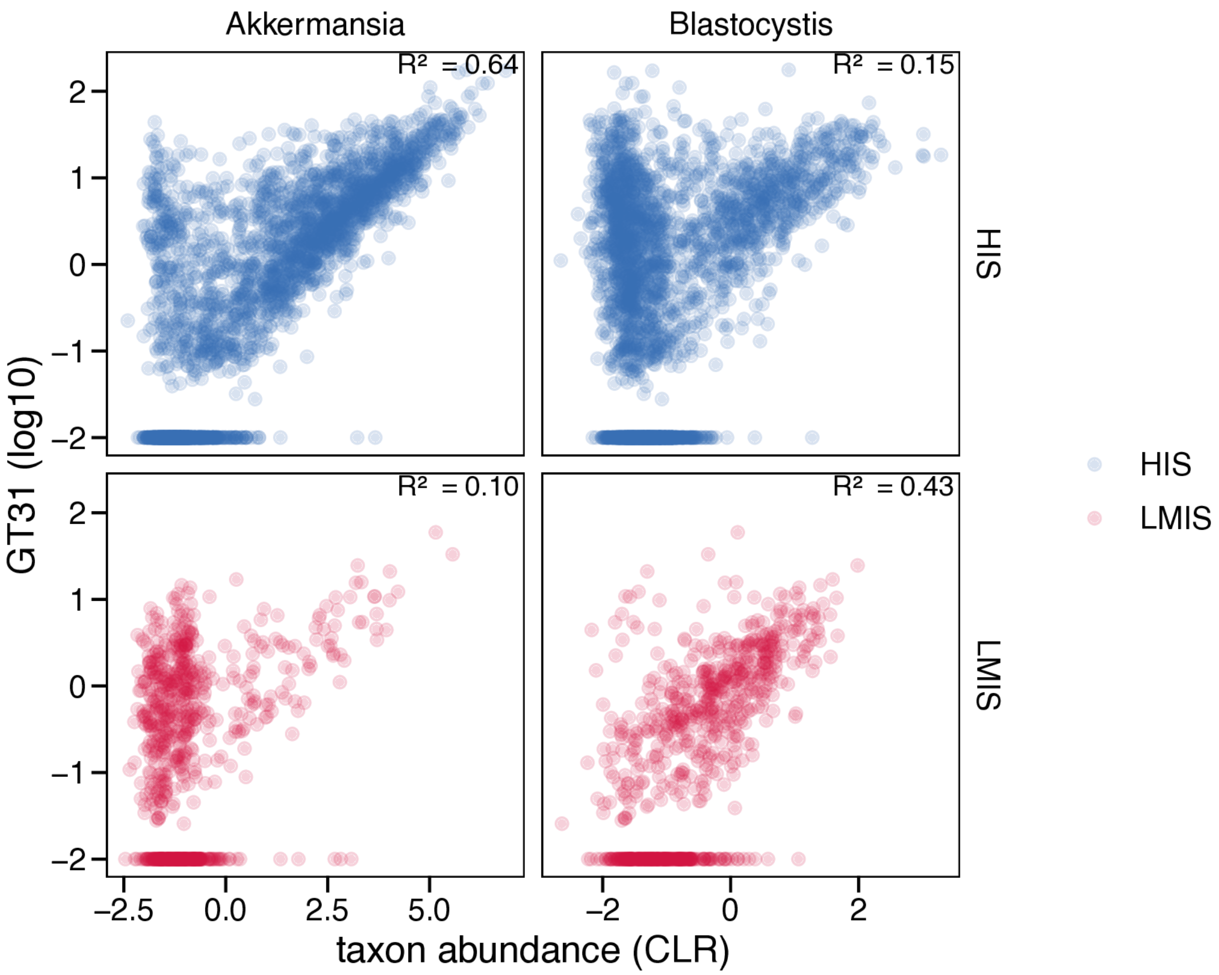

Supplementary Figure 3: Scatter plots between Akkermansia/Blastocystis abundances (Centered-log-ratio-scaled) and GT31 abundances (log10-scaled) for both HIS and LMIS groups. n = 3,166 for HIS and n = 794 for LMIS.
