# Supplementary material for: Cayman enables large-scale analysis of gut microbiome carbohydrate-active enzyme repertoires
Source: Nat Microbiol. 2026 Apr 24;11(6):1739–53. doi: 10.1038/s41564-026-02318-2 (PMC13236585; doi:10.1038/s41564-026-02318-2)
Supplement: Supplementary file 3 — Peer Review File [file 41564_2026_2318_MOESM3_ESM.pdf]

nature portfolio

Peer Review File

Cayman enables large-scale analysis of gut microbiome CAZyme repertoires

Corresponding Author: Professor Georg Zeller

Version 0:

Reviewer comments:

Reviewer #1

(Remarks to the Author)
Ducarmon et al.

Large scale computational analyses of gut microbial CAZyme repertoires enabled by Cayman

Carbohydrate-active enzymes (CAZymes) are an important group of enzymes in the human gut microbiome involved in oligo- and polysaccharide metabolism of both plant and host origin. Given that humans almost completely lack the ability to metabolize such glycans, and given that the altered microbiome functions are associated with various diseases, bioinformatic analysis tools to detail out CAZyme composition in given metagenomic datasets will continue to be valuable for the field. One important existing resource, that is updated regularly, is CAZy (www.CAZy.org), which describes which enzymes target which substrates. However, to infer substrates from large metagenomics datasets and to expedite reporting of CAZyme compositions would require development of additional bioinformatics tools. Thus, the authors developed a scalable command line tool (Cayman), with the addition of a manually curated database of CAZyme substrates, which would streamline the process of detecting biologically meaningful CAZyme shifts in the gut microbiome metagenomic datasets. No new data were generated and the authors used existing metagenomic datasets from public repositories to test their tool. Overall, this is an interesting concept. The authors used their tool and substrate annotation: 1) to explore the overall CAZyme repertoires of gut bacteria using The Genome Taxonomy Database (GDTB) database; and 2) to examine the CAZyme repertoire in the context of Western and non-Western diet and colorectal cancer. The methods and analysis are easy to follow. However, there are a number of concerns that challenge the novelty and broader applicability of this study, which altogether dampen the overall enthusiasm about this study. See seven major reservations about this study outlined below.
NB: with regards to the assessment of the scalability, and ease to use Cayman, hopefully, this can be better judged by bioinformatics specialists (other reviewers).

Hierachical substrate annotation:
1) The authors note in the abstract that "interpretation of substrate preference is lacking". The recent publication of DBCan3 in May 2023, which authors properly cite, contains a manually curated database of CAZYme substrates on the CAZyme subfamily level (link to the sheets: https://bcb.unl.edu/dbCAN2/download/Databases/dbCAN-sub.substrate.mapping.xls), which are surprisingly similar efforts compared to the current approach of the authors. Additionally, the dbCAN3 curated table contains a higher grouping of mucin and host-glycan (although groupings of dietary fibers are missing). Did the authors use this table as a resource during substrate annotation? What is the overlap between their annotation and one in dbCAN3? dbCAN3 is updated annually and is well maintained. How do authors plan to address this practical point? Moreover, how does the usage of dbCAN2 version 2020 relate to making the Cayman tool up to date? How many CAZymes have been added to the database in the meantime? Finally, dbCAN3 provides a substrate prediction tool. Could this be incorporated with the Cayman to extend the scope of analysis to less studied species and increase the overall knowledge of substrate specificity in downstream analysis?
2) Although authors justify their hierarchical grouping of CAZymes as potentially biologically meaningful based on a publication (reference 17), cases presented in the publication only use group 1 hierarchy (glycan substrate grouping). Since the authors did not provide any use cases Group 2 and Group 3 functional annotation, the overall biological utility of such classification cannot be judged. Therefore, biological relevance and the claim that “These can be used for interpretation of downstream statistical analysis at various levels of granularity” (lines 111-112) cannot be fully confirmed based on the presented analysis. Additionally, due to the lack of experimental knowledge, many CAZymes entries from Groups 2 and 3 are unknown, which limits the usability of such a system of annotation at the current state. This also raises a question about the timely maintenance of Cayman.

Exploration of CAZymes in human gut microbiomes:
3) By leveraging Cayman, the authors confirm a previously described capacity of glycan degradation in Bacteroides and Parabacteroides genera, while highlighting Hungatella and Eisenbergiella genera from Firmicutes phylum, as Firmicutes genera are particularly rich in CAZyme repertoire. However, the sentence "but to our knowledge, neither genus has been experimentally studied for their glycan utilization capabilities“ (lines 147-149) is not completely true. Rawat et al. (doi: 10.1128/aem.01546-22)

studied the growth of 4 glycosaminoglycans of an isolate of Hungatella Hathweyi. Interestingly, in the same publication, in Figure 1 by using a similar approach (HHMER with dbCAN2), they report an abundance of GAG Cazymes in Eisenbergiella spp, as well as in some other Firmicutes.
4) Moreover, substrate specificity analysis outlined Eisnbergiella and Coprobacter as promising mucin degraders. An experimental validation of such findings derived from Cayman is essential, as this would serve as a proof- of-concept for the genetic prediction of substrate annotation curated by the authors. For example, growing the type strains of both isolates on minimal media with purified mucin O-glycans and showing with transcriptomic analysis that indeed the identified CAZymes are involved in mucin degradation. Without such validation, the ambiguity in labelling a strain as a mucin degrader would not be pacified.
5) Lastly, the claim "Taken together, our analysis gives a systematic overview of the carbohydrate-related metabolic potential of human gut bacteria " (lines 177-179) needs to be backed up by more in-depth analysis. We agree with the authors that their analysis is more comprehensive compared to previous reports (10.1038/nrmicro3050) and we generally find Figure 2a quite a useful overview of CAZyme abundance and broad substrate specificity. To add additional value to the overview, the species-level diversity in the CAZyme repertoire should be explored (possibly as extended data figure(s) and a table that could be used as a resource). As an illustrative example of why such information is necessary: just by looking at Figure 2, Ruminococcus genus is not enriched in CAZyme repertoire for mucin degradation but we know that Ruminococcus gnavus (DOI: 10.1093/femsre/fuad014) and Rumonococcus torques (DOIs: 10.1101/2024.01.15.575725, 10.1172/JCI111795) are prominent mucin degraders in human gut. This existing literature raises an important question that a genus-level analysis might obscure potential diversity in the overall CAZyme richness as well as diversity in substrate specificity within the genus (i.e. on a species level). Therefore, showing which species contribute most CAZyme enriches would provide a more in-depth systematic overview that could be used as a resource by other researchers.
6) Importantly, we would like to point out that there is striking, yet underexplored strain level diversity in glycan degrading capabilities (especially mucin and glycosaminoglycan) in some species, as experimentally proven in Pudlo et al and Porras et al. (correctly referenced by authors at 7 & 20). As an illustrative example, Bacteroidetes ovatus strain has high diversity in the capability of growth on mucin-o-glycans (see Pudlo et al). This has to be addressed in the discussion of the results and the overall utility of genetic-phenotype prediction.

CAZyme capability in the context of Western and non-wester diets and colorectal cancer:
7) The analysis revealed a surprising finding of an interesting high abundance of CAZyme in the Western diet and enriched in mucin and GAG Cazymes. An additional analysis properly excluded the possibility that the finding is solely emerging from the difference in reading mappings. The authors properly addressed the potential reasons for these findings in the discussion. However, the authors need to take on board the comments made above regarding Exploration of CAZymes in human gut microbiomes.

Minor point:
Line (111-113) – The example of CBM75 is not backed up in figure 1A as referenced and might confuse the reader.

Reviewer #2

(Remarks to the Author)
This is a large body of work and a significant analysis of the existing microbial data. The correlations it brings out are incredibly interesting and are of value to a broad community not just CAZymologists. It would have been nice, however, if even just some simple wet experiments had been done to prove the utility of the software, i.e. growing suspected O-glycan degraders on O-glycans. This is a simple experiment that could be conducted in collaboration if the authors did not have the facilities.

I think the potential impact of the Cayman needs to be brought out more in the introduction and how it exceeds the limitations of what is currently available. It was not immediately clear to the size in the step the authors were claiming. It did seem to come through more in the discussion.

There needs to be more careful analysis of the literature and what the CAZymes actually do and what is known about their biochemistry. I have made specific comments about GH29 and GH95, and GHs used in Figure 2B to imply mucin foraging. There is also no analysis of carbohydrate sulfatases which have been shown to be important for host glycan metabolism.

All in all the work looks strong and is interesting but is a large number of very interesting correlations that need to be phenotypically tested. Maybe the power is in the software can be accessed and deployed by wet labs, but how easy that will be for novices I am unsure, but the work is clearly of value.

Specific comments

Lines 67-71
CAzy is a database cataloguing the literature so the limitations here are clear but dbCAN can annotate genomes and predict active as it says in the header on the webage, what limitation does dbCAN have? Is it only one genome at a time? Can it not handle metagenomic data? There is also no mention of PULDB which is a useful resource for predicting enzyme function/substrate target by PUL composition. Is the issue these are not open source?

Line 78-86
The issue I have here is the phrase 'CAZyme families into higher-level, biologically meaningful substrate groups' this must be done using data from the literature and CAZy, how else will Cayman predict what these enzymes are doing? When you have a PUL it can be easy to attempt to infer the target but when there is no PUL organisation in an organism simply ascribing its

substrate preferences based on CAZymes that are distally located on the genome is a risky business, especially if the software is used by non CAZymologists.

Line 99 Developing and curating a CAZyme substrate scheme
How is the example of CBM75 helpful? There is only 1 sequence on CAZy and the CBM is appended to GH43_16 which are arabinofuranosidases, xylosidases, and xylanases.

Line146-147
Among these, Hungatella and Eisenbergiella stood out as distinctly rich in CAZymes (with
224 genes from 78 families and 404 genes from 106 families, respectively),
This is a remarkable finding but it would be good to see these genes listed somewhere in the supplemental. Saying the Bacteroides have high numbers and the Firmicutes (now Bacillota) have less is well known in the field. The data that two Bacillota organisms, that have not been well studied, have huge numbers needs more explicit data than just a bar on the bar chart if others are to use the data as a springboard.

Figure 2B
I think there needs to some caution about calling all the CAZyme family selected as mucin related:

GH2 is a polyspecific family working on a vast number of substrates both host and plant

GH31 and GH35: These both have members working on plant and host glycans has this been accounted for?

GH29 and GH95: within both these families the presence of Thr or His in the active site correlates with if the enzymes are L-fucosidases or L-galactosidases. L-galactose is no found in mucin and any sequences with targeting this would need to be removed. In addition, plant glycans also contain L-fucose (refs: https://doi.org/10.1038/ncomms8481 and doi: 10.1038/s41589-022-00983-y_

GH84 is infamous as being an O-GlcNAcase removing GlcNAc from phosphorylation sites.

GH108 the only annotation on CAZy is lysozyme? This would mean it targets peptidoglycans?

GH130 has members specific for fungal beta mannans

Lines 167-174
The inferences are nice, and make great hypotheses, but it would have been better to show that some of these organisms are specialist mucin degraders. At the moment this is a correlation and not proof.

Line 173
genomic copies for several mucin-targeting GHs, including α-fucosidases (GH29 and GH95),
The absence of presence of Thr or His in the base of the -1 subsite needs checking, and L-galactosidases discounted.

Line 261
On the other hand, the most Western-enriched CAZyme, CBM58, is part of the SusG protein
strictly speaking CBMs are not CAZymes (Carbohydrate active enzymes), but I am not sure if there is a catch all term alternative that covers CBMs (SGBPs are also not CAZymes).

Figure 4 D again it would be interesting to see the polymorphism spread in the GH95s and how this maps to the data.

Line 306 is Fig 4D at the start right as everything is about figure 4E and F.

Line 314 Fig 4F call out should be 4G

Line 365-370
Taken together, these results showed a clear enrichment of
CAZymes involved in host- and animal glycan metabolism in CRC metagenomes
I would if the authors considered using sulfatases as an important CAZyme? They are essential enzymes for the degradation of sulfated host glycans in the colon (GAGs and MUC2), and would provide stronger inferences to their data.

Lines 388-400
CBMs often mirror the activity of the enzyme they are appended too but not always, sometimes they bind an abundant glycan that is close to the target of the enzyme. Plant cell wall degrading xylanases have been shown to have cellulose binding CBMs. The ‘guilt by association’ is not novel to this paper and known CBMs appended to large genes of unknown function have been used as probes to find new enzymes functions previously.

Reviewer #4

(Remarks to the Author)
Summary -
In this manuscript, the authors describe their new Cayman tool for profiling CAZymes and annotating their preferred substrates in metagenomic data. Their hierarchical substrate annotation scheme will enable researchers to more confidently assign functions

to specific CAZymes. The authors deploy their tool to look for differences in CAZyme substrate preferences between industrialized and non-industrialized populations and between patients with colorectal cancer and healthy patients. The results of these analyses are intuitive, and provide confidence in the tool. Overall, the tool is useful, the methodology is appropriate, the figures are informative, and the manuscript is well written. I commend the authors on a great tool and a great manuscript. I don't have any major concerns with the work. Below I provide some minor comments to help improve the presentation.

Specific Comments -

The Western/Non-Western dichotomy is Euro-centrist language, which is falling more and more out of fashion. I often find it confusing when countries like Japan, Australia, and South Korea are labeled with 'Western' lifestyles. It seems more precise to use dichotomies like: Developed/Developing, Industrialized/Non-industrialized, Urban/Rural, and High vs. Low and Middle Income. To avoid sounding dated in a few years, I suggest using more precise language, when it comes to the exact populations included in your first meta-analysis.

I often found myself getting lost in the acronyms, and certain acronyms didn't appear to be defined (dbCAN, pHMMs). Please double check that you've defined them, and perhaps make a little table that summarizes these acronyms for the reader.

line 24: Maybe change 'richer' to 'enriched'?

line 27: Suggest changing 'deprived of' to 'depleted in'

line 60: suggest replacing 'the stark contrast has been instrumental to uncover shifts of gut' to 'the contrast has uncovered shifts in key'

line 567: change 'on based' to 'based on'

Decision Letter:

1st May 2024

*Please ensure you delete the link to your author homepage in this e-mail if you wish to forward it to your co-authors.

Dear Dr Zeller,

Thank you for your patience while your manuscript "Large-scale computational analyses of gut microbial CAZyme repertoires enabled by Cayman" was under peer-review at Nature Microbiology, and please accept my apologies for the delay in getting back to you with a decision. It has now been seen by 3 referees, whose expertise and comments you will find at the end of this email. Although they find your work of some potential interest, they have raised a number of concerns that will need to be addressed before we can consider publication of the work in Nature Microbiology.

In particular,Reviewers 1 and 2 feel a better comparison with dbCAN should be provided to highlight the advantages of Cayman. They both feel experimental validations and follow up of a couple of the findings would benefit the work, which we ask you to address.

Should further experimental data allow you to address these criticisms, we would be happy to look at a revised manuscript.

We are committed to providing a fair and constructive peer-review process. Please do not hesitate to contact us if there are specific requests from the reviewers that you believe are technically impossible or unlikely to yield a meaningful outcome.

We strongly support public availability of data. Please place the data used in your paper into a public data repository, if one exists, or alternatively, present the data as Source Data or Supplementary Information. If data can only be shared on request, please explain why in your Data Availability Statement, and also in the correspondence with your editor. For some data types, deposition in a public repository is mandatory - more information on our data deposition policies and available repositories can be found at https://www.nature.com/nature-research/editorial-policies/reporting-standards#availability-of-data.

Please include a data availability statement as a separate section after Methods but before references, under the heading "Data Availability". This section should inform readers about the availability of the data used to support the conclusions of your study. This information includes accession codes to public repositories (data banks for protein, DNA or RNA sequences, microarray, proteomics data etc…), references to source data published alongside the paper, unique identifiers such as URLs to data repository entries, or data set DOIs, and any other statement about data availability. At a minimum, you should include the following statement: “The data that support the findings of this study are available from the corresponding author upon request”, mentioning any restrictions on availability. If DOIs are provided, we also strongly encourage including these in the Reference list (authors, title, publisher (repository name), identifier, year). For more guidance on how to write this section please see: http://www.nature.com/authors/policies/data/data-availability-statements-data-citations.pdf

If revising your manuscript:

* Include a “Response to referees” document detailing, point-by-point, how you addressed each referee comment. If no action was taken to address a point, you must provide a compelling argument. This response will be sent back to the referees along

with the revised manuscript.

* If you have not done so already we suggest that you begin to revise your manuscript so that it conforms to our Article format instructions at http://www.nature.com/nmicrobiol/info/final-submission. Refer also to any guidelines provided in this letter.

* Include a revised version of any required reporting checklist. It will be available to referees (and, potentially, statisticians) to aid in their evaluation if the manuscript goes back for peer review. A revised checklist is essential for re-review of the paper.

When submitting the revised version of your manuscript, please pay close attention to our href="https://www.nature.com/nature-portfolio/editorial-policies/image-integrity">Digital Image Integrity Guidelines. and to the following points below:

-- that unprocessed scans are clearly labelled and match the gels and western blots presented in figures.
-- that control panels for gels and western blots are appropriately described as loading on sample processing controls
-- all images in the paper are checked for duplication of panels and for splicing of gel lanes.

Finally, please ensure that you retain unprocessed data and metadata files after publication, ideally archiving data in perpetuity, as these may be requested during the peer review and production process or after publication if any issues arise.

Please use the link below to submit a revised paper:

Link Redacted

Note: This url links to your confidential homepage and associated information about manuscripts you may have submitted or be reviewing for us. If you wish to forward this e-mail to co-authors, please delete this link to your homepage first.

Nature Microbiology is committed to improving transparency in authorship. As part of our efforts in this direction, we are now requesting that all authors identified as 'corresponding author' on published papers create and link their Open Researcher and Contributor Identifier (ORCID) with their account on the Manuscript Tracking System (MTS), prior to acceptance. This applies to primary research papers only. ORCID helps the scientific community achieve unambiguous attribution of all scholarly contributions. You can create and link your ORCID from the home page of the MTS by clicking on 'Modify my Springer Nature account'. For more information please visit please visit www.springernature.com/orcid.

If you wish to submit a suitably revised manuscript we would hope to receive it within 6 months. If you cannot send it within this time, please let us know. We will be happy to consider your revision, even if a similar study has been accepted for publication at Nature Microbiology or published elsewhere (up to a maximum of 6 months).

In the meantime we hope that you find our referees' comments helpful.

Yours sincerely,

****
Reviewer Expertise:

Referee #1: CAZymes
Referee #2: CAZymes
Referee #3: Bioinformatics

Reviewer Comments:

Reviewer #1 (Remarks to the Author):

Ducarmon et al.

Large scale computational analyses of gut microbial CAZyme repertoires enabled by Cayman

Carbohydrate-active enzymes (CAZymes) are an important group of enzymes in the human gut microbiome involved in oligo- and polysaccharide metabolism of both plant and host origin. Given that humans almost completely lack the ability to metabolize such glycans, and given that the altered microbiome functions are associated with various diseases, bioinformatic analysis tools to detail out CAZyme composition in given metagenomic datasets will continue to be valuable for the field. One important existing resource, that is updated regularly, is CAZy (www.CAZy.org), which describes which enzymes target which substrates. However, to infer substrates from large metagenomics datasets and to expedite reporting of CAZyme compositions would require development of additional bioinformatics tools. Thus, the authors developed a scalable command line tool (Cayman), with the addition of a manually curated database of CAZyme substrates, which would streamline the process of detecting biologically meaningful CAZyme shifts in the gut microbiome metagenomic datasets. No new data were generated and the authors used existing metagenomic datasets from public repositories to test their tool. Overall, this is an interesting concept. The authors used their tool and substrate annotation: 1) to explore the overall CAZyme repertoires of gut bacteria using The Genome

Taxonomy Database (GDTB) database; and 2) to examine the CAZyme repertoire in the context of Western and non-Western diet and colorectal cancer. The methods and analysis are easy to follow. However, there are a number of concerns that challenge the novelty and broader applicability of this study, which altogether dampen the overall enthusiasm about this study. See seven major reservations about this study outlined below.
NB: with regards to the assessment of the scalability, and ease to use Cayman, hopefully, this can be better judged by bioinformatics specialists (other reviewers).

Hierachical substrate annotation:
1) The authors note in the abstract that "interpretation of substrate preference is lacking". The recent publication of DBCan3 in May 2023, which authors properly cite, contains a manually curated database of CAZYme substrates on the CAZyme subfamily level (link to the sheets: https://bcb.unl.edu/dbCAN2/download/Databases/dbCAN-sub.substrate.mapping.xls), which are surprisingly similar efforts compared to the current approach of the authors. Additionally, the dbCAN3 curated table contains a higher grouping of mucin and host-glycan (although groupings of dietary fibers are missing). Did the authors use this table as a resource during substrate annotation? What is the overlap between their annotation and one in dbCAN3? dbCAN3 is updated annually and is well maintained. How do authors plan to address this practical point? Moreover, how does the usage of dbCAN2 version 2020 relate to making the Cayman tool up to date? How many CAZymes have been added to the database in the meantime? Finally, dbCAN3 provides a substrate prediction tool. Could this be incorporated with the Cayman to extend the scope of analysis to less studied species and increase the overall knowledge of substrate specificity in downstream analysis?
2) Although authors justify their hierarchical grouping of CAZymes as potentially biologically meaningful based on a publication (reference 17), cases presented in the publication only use group 1 hierarchy (glycan substrate grouping). Since the authors did not provide any use cases Group 2 and Group 3 functional annotation, the overall biological utility of such classification cannot be judged. Therefore, biological relevance and the claim that “These can be used for interpretation of downstream statistical analysis at various levels of granularity” (lines 111-112) cannot be fully confirmed based on the presented analysis. Additionally, due to the lack of experimental knowledge, many CAZymes entries from Groups 2 and 3 are unknown, which limits the usability of such a system of annotation at the current state. This also raises a question about the timely maintenance of Cayman.

Exploration of CAZymes in human gut microbiomes:
3) By leveraging Cayman, the authors confirm a previously described capacity of glycan degradation in Bacteroides and Parabacteroides genera, while highlighting Hungatella and Eisenbergiella genera from Firmicutes phylum, as Firmicutes genera are particularly rich in CAZyme repertoire. However, the sentence "but to our knowledge, neither genus has been experimentally studied for their glycan utilization capabilities“ (lines 147-149) is not completely true. Rawat et al. (doi: 10.1128/aem.01546-22) studied the growth of 4 glycosaminoglycans of an isolate of Hungatella Hathweyi. Interestingly, in the same publication, in Figure 1 by using a similar approach (HHMER with dbCAN2), they report an abundance of GAG Cazymes in Eisenbergiella spp, as well as in some other Firmicutes.
4) Moreover, substrate specificity analysis outlined Eisnbergiella and Coprobacter as promising mucin degraders. An experimental validation of such findings derived from Cayman is essential, as this would serve as a proof- of-concept for the genetic prediction of substrate annotation curated by the authors. For example, growing the type strains of both isolates on minimal media with purified mucin O-glycans and showing with transcriptomic analysis that indeed the identified CAZymes are involved in mucin degradation. Without such validation, the ambiguity in labelling a strain as a mucin degrader would not be pacified.
5) Lastly, the claim "Taken together, our analysis gives a systematic overview of the carbohydrate-related metabolic potential of human gut bacteria “ (lines 177-179) needs to be backed up by more in-depth analysis. We agree with the authors that their analysis is more comprehensive compared to previous reports (10.1038/nrmicro3050) and we generally find Figure 2a quite a useful overview of CAZyme abundance and broad substrate specificity. To add additional value to the overview, the species-level diversity in the CAZyme repertoire should be explored (possibly as extended data figure(s) and a table that could be used as a resource). As an illustrative example of why such information is necessary: just by looking at Figure 2, Ruminococcus genus is not enriched in CAZyme repertoire for mucin degradation but we know that Ruminococcus gnavus (DOI: 10.1093/femsre/fuad014) and Rumonococcus torques (DOIs: 10.1101/2024.01.15.575725, 10.1172/JCI111795) are prominent mucin degraders in human gut. This existing literature raises an important question that a genus-level analysis might obscure potential diversity in the overall CAZyme richness as well as diversity in substrate specificity within the genus (i.e. on a species level). Therefore, showing which species contribute most CAZyme enriches would provide a more in-depth systematic overview that could be used as a resource by other researchers.
6) Importantly, we would like to point out that there is striking, yet underexplored strain level diversity in glycan degrading capabilities (especially mucin and glycosaminoglycan) in some species, as experimentally proven in Pudlo et al and Porras et al. (correctly referenced by authors at 7 & 20). As an illustrative example, Bacteroidetes ovatus strain has high diversity in the capability of growth on mucin-o-glycans (see Pudlo et al). This has to be addressed in the discussion of the results and the overall utility of genetic-phenotype prediction.

CAZyme capability in the context of Western and non-wester diets and colorectal cancer:
7) The analysis revealed a surprising finding of an interesting high abundance of CAZyme in the Western diet and enriched in mucin and GAG Cazymes. An additional analysis properly excluded the possibility that the finding is solely emerging from the difference in reading mappings. The authors properly addressed the potential reasons for these findings in the discussion. However, the authors need to take on board the comments made above regarding Exploration of CAZymes in human gut microbiomes.

Minor point:
Line (111-113) – The example of CBM75 is not backed up in figure 1A as referenced and might confuse the reader.

Reviewer #2 (Remarks to the Author):

This is a large body of work and a significant analysis of the existing microbial data. The correlations it brings out are incredibly interesting and are of value to a broad community not just CAZymologists. It would have been nice, however, if even just some simple wet experiments had been done to prove the utility of the software, i.e. growing suspected O-glycan degraders on O-glycans. This is a simple experiment that could be conducted in collaboration if the authors did not have the facilities.

I think the potential impact of the Cayman needs to be brought out more in the introduction and how it exceeds the limitations of what is currently available. It was not immediately clear to the size in the step the authors were claiming. It did seem to come through more in the discussion.

There needs to be more careful analysis of the literature and what the CAZymes actually do and what is known about their biochemistry. I have made specific comments about GH29 and GH95, and GHs used in Figure 2B to imply mucin foraging. There is also no analysis of carbohydrate sulfatases which have been shown to be important for host glycan metabolism.

All in all the work looks strong and is interesting but is a large number of very interesting correlations that need to be phenotypically tested. Maybe the power is in the software can be accessed and deployed by wet labs, but how easy that will be for novices I am unsure, but the work is clearly of value.

Specific comments

Lines 67-71
CAzy is a database cataloguing the literature so the limitations here are clear but dbCAN can annotate genomes and predict active as it says in the header on the webage, what limitation does dbCAN have? Is it only one genome at a time? Can it not handle metagenomic data? There is also no mention of PULDB which is a useful resource for predicting enzyme function/substrate target by PUL composition. Is the issue these are not open source?

Line 78-86
The issue I have here is the phrase 'CAZyme families into higher-level, biologically meaningful substrate groups' this must be done using data from the literature and CAZy, how else will Cayman predict what these enzymes are doing? When you have a PUL it can be easy to attempt to infer the target but when there is no PUL organisation in an organism simply ascribing its substrate preferences based on CAZymes that are distally located on the genome is a risky business, especially if the software is used by non CAZymologists.

Line 99 Developing and curating a CAZyme substrate scheme
How is the example of CBM75 helpful? There is only 1 sequence on CAZy and the CBM is appended to GH43_16 which are arabinofuranosidases, xylosidases, and xylanases.

Line146-147
Among these, Hungatella and Eisenbergiella stood out as distinctly rich in CAZymes (with
224 genes from 78 families and 404 genes from 106 families, respectively),
This is a remarkable finding but it would be good to see these genes listed somewhere in the supplemental. Saying the Bacteroides have high numbers and the Firmicutes (now Bacillota) have less is well known in the field. The data that two Bacillota organisms, that have not been well studied, have huge numbers needs more explicit data than just a bar on the bar chart if others are to use the data as a springboard.

Figure 2B
I think there needs to some caution about calling all the CAZyme family selected as mucin related:

GH2 is a polyspecific family working on a vast number of substrates both host and plant

GH31 and GH35: These both have members working on plant and host glycans has this been accounted for?

GH29 and GH95: within both these families the presence of Thr or His in the active site correlates with if the enzymes are L-fucosidases or L-galactosidases. L-galactose is no found in mucin and any sequences with targeting this would need to be removed. In addition, plant glycans also contain L-fucose (refs: https://doi.org/10.1038/ncomms8481 and doi: 10.1038/s41589-022-00983-y_

GH84 is infamous as being an O-GlcNAcase removing GlcNAc from phosphorylation sites.

GH108 the only annotation on CAZy is lysozyme? This would mean it targets peptidoglycans?

GH130 has members specific for fungal beta mannans

Lines 167-174
The inferences are nice, and make great hypotheses, but it would have been better to show that some of these organisms are specialist mucin degraders. At the moment this is a correlation and not proof.

Line 173
genomic copies for several mucin-targeting GHs, including α-fucosidases (GH29 and GH95),
The absence of presence of Thr or His in the base of the -1 subsite needs checking, and L-galactosidases discounted.

Line 261
On the other hand, the most Western-enriched CAZyme, CBM58, is part of the SusG protein
strictly speaking CBMs are not CAZymes (Carbohydrate active enzymes), but I am not sure if there is a catch all term alternative that covers CBMs (SGBPs are also not CAZymes).

Figure 4 D again it would be interesting to see the polymorphism spread in the GH95s and how this maps to the data.

Line 306 is Fig 4D at the start right as everything is about figure 4E and F.

Line 314 Fig 4F call out should be 4G

Line 365-370
Taken together, these results showed a clear enrichment of
CAZymes involved in host- and animal glycan metabolism in CRC metagenomes
I would if the authors considered using sulfatases as an important CAZyme? They are essential enzymes for the degradation of sulfated host glycans in the colon (GAGs and MUC2), and would provide stronger inferences to their data.

Lines 388-400
CBMs often mirror the activity of the enzyme they are appended too but not always, sometimes they bind an abundant glycan that is close to the target of the enzyme. Plant cell wall degrading xylanases have been shown to have cellulose binding CBMs. The ‘guilt by association’ is not novel to this paper and known CBMs appended to large genes of unknown function have been used as probes to find new enzymes functions previously.

Reviewer #3 (Remarks to the Author):

Summary -
In this manuscript, the authors describe their new Cayman tool for profiling CAZymes and annotating their preferred substrates in metagenomic data. Their hierarchical substrate annotation scheme will enable researchers to more confidently assign functions to specific CAZymes. The authors deploy their tool to look for differences in CAZyme substrate preferences between industrialized and non-industrialized populations and between patients with colorectal cancer and healthy patients. The results of these analyses are intuitive, and provide confidence in the tool. Overall, the tool is useful, the methodology is appropriate, the figures are informative, and the manuscript is well written. I commend the authors on a great tool and a great manuscript. I don't have any major concerns with the work. Below I provide some minor comments to help improve the presentation.

Specific Comments -

The Western/Non-Western dichotomy is Euro-centrist language, which is falling more and more out of fashion. I often find it confusing when countries like Japan, Australia, and South Korea are labeled with 'Western' lifestyles. It seems more precise to use dichotomies like: Developed/Developing, Industrialized/Non-industrialized, Urban/Rural, and High vs. Low and Middle Income. To avoid sounding dated in a few years, I suggest using more precise language, when it comes to the exact populations included in your first meta-analysis.

I often found myself getting lost in the acronyms, and certain acronyms didn't appear to be defined (dbCAN, pHMMs). Please double check that you've defined them, and perhaps make a little table that summarizes these acronyms for the reader.

line 24: Maybe change 'richer' to 'enriched'?

line 27: Suggest changing 'deprived of' to 'depleted in'

line 60: suggest replacing 'the stark contrast has been instrumental to uncover shifts of gut' to 'the contrast has uncovered shifts in key'

line 567: change 'on based' to 'based on'

****

Version 2:

Reviewer comments:

Reviewer #1

(Remarks to the Author)
I thank the authors for their revisions; the manuscript is substantially improved. Cayman will be an important resource for the

community.

Minor point: Line 544 word "better" duplicated

(Remarks on code availability)

Reviewer #5

(Remarks to the Author)

(Remarks on code availability)

Reviewer #6

(Remarks to the Author)
The paper by Ducarmon et al describes the development of an automated tool for annotating metagenomic data with CAZyme families and predicted substrates targeted. This would indeed be a useful addition to the field as this currently can't be done with metagenomic data leading to problems with enzyme annotation and subsequent predictions of potential substrates targeted. The authors then demonstrate the utility of their tool by identifying two novel mucin users from the human gut microbiota.
My expertise isn't in bioinformatics so I couldn't install Cayman to test, but hopefully the program is easy to use as the authors indicate – all of my comments are main about some of the inferences made in relation to the glycans targeted by the human gut microbiota.
1. Fig 1A. Starch – only resistant starch is classified as dietary fibre. This should be highlighted.
2. Fiig 1B. While GAGs will be present in meat, they will also likely be in large amounts in the intestine from sloughed epithelial cells (both small and large intestine have significant normal cell turnover). This should also be noted.
3. Fig 6C. Care should be taken in assigning cellulose as a target for the human gut microbiota (see also line 430). Cellulose breakdown in the human gut is thought to be limited, esp in modern industrialised societies (see e.g. PMID: 38484069). This is due to lack of dedicated cellulose degraders, but also likely the fast transit time of the human gut vs dedicated herbivores. Cooking food can obviously influence the accessibility of cellulose, but I'm not aware of any studies where dedicated insoluble cellulose degrading apparatus has been characterised in human gut microbes. If these exist please reference the studies here.
4. Fig 6C. mannans and heteromannans. Are these plant b-mannans (cell wall or storage polysaccharides), or alpha mannans as found in the fungal cell wall? (see e.g PMID: 31649168). Does Cayman distinguish between these?
5. Fig 2c – shouldn't Akkermansia light up as a red dot in the mucin colour coded box? Seems to be only Bacteroides spp that light up red in all (DF, mucin, GAGs)?
6. Fig 4d, legend - line 268 states 'dots indicate outliers' – I can't see any dots on Fig 4d?
7. Lines 373-376 states that Akkermansia is the principal carrier of GT31 in HIS, but not LMIS individuals. From Cazy GT31 appears to be only found in Akkermansia (in bacteria). So is GT31 lacking in LMIS or carried by another taxa? Or is it because Akkermansia are rare in LMIS individuals? (see e.g. PMID: 31649168).
8. Lines 466-468 – I don't think can claim that the analysis presented here 'revealed' points ii and iii – both are well known features of CBM-GH associations.
Typo – line 544 'better' written twice

(Remarks on code availability)

Decision Letter:

5th December 2025

*Please ensure you delete the link to your author homepage in this e-mail if you wish to forward it to your co-authors.

Dear Georg,

Thank you for your patience while your manuscript "Large-scale computational analyses of gut microbial CAZyme repertoires enabled by Cayman" was under peer-review at Nature Microbiology. It has now been seen by 3 referees, whose expertise and comments you will find at the of this email. You will see from their comments below that while they find your work of interest, some important points are raised. We are very interested in the possibility of publishing your study in Nature Microbiology, but would like to consider your response to these concerns in the form of a revised manuscript before we make a final decision on publication.

I'm sorry for the extended delay but we had some highly unusual issues with referees. Our original CAZyme referee was unresponsive and unfortunately our replacement referee (R4) could not submit a full report due to medical issues. Their partial review is appended below. I therefore recruited a final referee (R5) with these expertise to provide an assessment of your revisions.

In particular, you will see that overall the referees are happy and only relatively minor text edits are required. The rest referees'

reports are clear and the remaining issues should be straightforward to address. We will be unlikely to go back to referees once these revisions have been implemented.

We are committed to providing a fair and constructive peer-review process. Do not hesitate to contact us if there are specific requests from the reviewers that you believe are technically impossible or unlikely to yield a meaningful outcome.

If you have not done so already please begin to revise your manuscript so that it conforms to our Resource format instructions at http://www.nature.com/nmicrobiol/info/final-submission/

The usual length limit for a Nature Microbiology Article is six display items (figures or tables) and 3,000 words. We have some flexibility, and can allow a revised manuscript at 3,500 words, but please consider this a firm upper limit. There is a trade-off of ~250 words per display item, so if you need more space, you could move a Figure or Table to Supplementary Information.

Some reduction could be achieved by focusing any introductory material and moving it to the start of your opening 'bold' paragraph, whose function is to outline the background to your work, describe in a sentence your new observations, and explain your main conclusions. The discussion should also be limited. Methods should be described in a separate section following the discussion, we do not place a word limit on Methods.

Nature Microbiology titles should give a sense of the main new findings of a manuscript, and should not contain punctuation. Please keep in mind that we strongly discourage active verbs in titles, and that they should ideally fit within 90 characters each (including spaces).

We strongly support public availability of data. Please place the data used in your paper into a public data repository, if one exists, or alternatively, present the data as Source Data or Supplementary Information. If data can only be shared on request, please explain why in your Data Availability Statement, and also in the correspondence with your editor. For some data types, deposition in a public repository is mandatory - more information on our data deposition policies and available repositories can be found at https://www.nature.com/nature-research/editorial-policies/reporting-standards#availability-of-data.

Please include a data availability statement as a separate section after Methods but before references, under the heading "Data Availability". This section should inform readers about the availability of the data used to support the conclusions of your study. This information includes accession codes to public repositories (data banks for protein, DNA or RNA sequences, microarray, proteomics data etc…), references to source data published alongside the paper, unique identifiers such as URLs to data repository entries, or data set DOIs, and any other statement about data availability. At a minimum, you should include the following statement: "The data that support the findings of this study are available from the corresponding author upon request", mentioning any restrictions on availability. If DOIs are provided, we also strongly encourage including these in the Reference list (authors, title, publisher (repository name), identifier, year). For more guidance on how to write this section please see: http://www.nature.com/authors/policies/data/data-availability-statements-data-citations.pdf

To improve the accessibility of your paper to readers from other research areas, please pay particular attention to the wording of the paper's opening bold paragraph, which serves both as an introduction and as a brief, non-technical summary in about 150 words. If, however, you require one or two extra sentences to explain your work clearly, please include them even if the paragraph is over-length as a result. The opening paragraph should not contain references. Because scientists from other sub-disciplines will be interested in your results and their implications, it is important to explain essential but specialised terms concisely. We suggest you show your summary paragraph to colleagues in other fields to uncover any problematic concepts.

If your paper is accepted for publication, we will edit your display items electronically so they conform to our house style and will reproduce clearly in print. If necessary, we will re-size figures to fit single or double column width. If your figures contain several parts, the parts should form a neat rectangle when assembled. Choosing the right electronic format at this stage will speed up the processing of your paper and give the best possible results in print. We would like the figures to be supplied as vector files - EPS, PDF, AI or postscript (PS) file formats (not raster or bitmap files), preferably generated with vector-graphics software (Adobe Illustrator for example). Please try to ensure that all figures are non-flattened and fully editable. All images should be at least 300 dpi resolution (when figures are scaled to approximately the size that they are to be printed at) and in RGB colour format. Please do not submit Jpeg or flattened TIFF files. Please see also 'Guidelines for Electronic Submission of Figures' at the end of this letter for further detail.

Figure legends must provide a brief description of the figure and the symbols used, within 350 words, including definitions of any error bars employed in the figures.

When submitting the revised version of your manuscript, please pay close attention to our href="https://www.nature.com/nature-research/editorial-policies/image-integrity">Digital Image Integrity Guidelines. and to the following points below:

-- that unprocessed scans are clearly labelled and match the gels and western blots presented in figures.
-- that control panels for gels and western blots are appropriately described as loading on sample processing controls
-- all images in the paper are checked for duplication of panels and for splicing of gel lanes.

EXTENDED DATA FIGURES

When re-submitting your manuscript, please ensure that any supplementary figures and tables that are crucial to the manuscript's conclusions are converted into Extended Data figures and tables to increase visibility of these data. Extended Data figures and tables are online-only (present in the online PDF and full-text HTML versions of the paper), peer-reviewed display items that provide essential background to the article but are not included in the main article due to space constraints. A

maximum of ten Extended Data display items (figures and tables) is permitted.

Finally, please ensure that you retain unprocessed data and metadata files after publication, ideally archiving data in perpetuity, as these may be requested during the peer review and production process or after publication if any issues arise.

Please include a statement before the acknowledgements naming the author to whom correspondence and requests for materials should be addressed.

Finally, we require authors to include a statement of their individual contributions to the paper -- such as experimental work, project planning, data analysis, etc. -- immediately after the acknowledgements. The statement should be short, and refer to authors by their initials. For details please see the Authorship section of our joint Editorial policies at http://www.nature.com/authors/editorial_policies/authorship.html

When revising your paper:

* include a point-by-point response to any editorial suggestions and to our referees. Please include your response to the editorial suggestions in your cover letter, and please upload your response to the referees as a separate document.

* ensure it complies with our format requirements for Letters as set out in our guide to authors at www.nature.com/nmicrobiol/info/gta/

* state in a cover note the length of the text, methods and legends; the number of references; number and estimated final size of figures and tables

* resubmit electronically if possible using the link below to access your home page:

Link Redacted

*This url links to your confidential homepage and associated information about manuscripts you may have submitted or be reviewing for us. If you wish to forward this e-mail to co-authors, please delete this link to your homepage first.

Please ensure that all correspondence is marked with your Nature Microbiology reference number in the subject line.

Nature Microbiology is committed to improving transparency in authorship. As part of our efforts in this direction, we are now requesting that all authors identified as 'corresponding author' on published papers create and link their Open Researcher and Contributor Identifier (ORCID) with their account on the Manuscript Tracking System (MTS), prior to acceptance. This applies to primary research papers only. ORCID helps the scientific community achieve unambiguous attribution of all scholarly contributions. You can create and link your ORCID from the home page of the MTS by clicking on 'Modify my Springer Nature account'. For more information please visit please visit www.springernature.com/orcid.

We hope to receive your revised paper within three weeks. If you cannot send it within this time, please let us know.

We look forward to hearing from you soon.

Yours sincerely,

****

Reviewer Expertise:

Referee #1: microbiome, metabolism
Referee #4: CAZymes
Referee #5: CAZymes

Reviewers Comments:

Reviewer #1 (Remarks to the Author):

I thank the authors for their revisions; the manuscript is substantially improved. Cayman will be an important resource for the community.

Minor point: Line 544 word "better" duplicated

Reviewer #4:

As the previous reviewer 2 mention in is previously revision, the tool developed to predict the specificity of CAZymes is of scientific interest but this should be also address carefully if this is to be used by non CAZYmologists. Of relevance, the reviewer asked if the authors leverage the previous knowledge regarding the substrate recognition of GH95s to which the authors apparently dismiss as 'anecdoctal evidence for substrate differences'. In my opinion this is quite a misjudgment of wording regarding a previously published article (PMID: 26112186) where structural and biochemical data backs up the conclusions. The lack of understanding of such concepts by the authors raises questions regarding how this software can be applied to distinguish between activities in poly-specific families.
Additionally, both reviewer 1 and 2 request a experimental validation Hungatella and Eisenbergiella grow on mucin (here I would like to know that reviewer 1 make a point to suggest the growth on purified mucins. Although the authors grew the bacteria in a source of mucin, it has been established by now that commercially available gastric mucins are not a pure substrate and it is not similar to colonic mucins (PMID: 38947523). The commercially available mucins are highly contaminated with other sources of glycans (such as glycosaminoglycans) so the use of this substrate does not validate the claim that the bacteria grows on mucin.

Additional comments that require to be corrected:
- The reviewer 2 comment of the lack of analysis of bacterial sulfatases active on mucin. Although the authors introduced this information in the current manuscript the inclusion of the analysis of S1_46 sulfates as a family active on O-glycans is incorrect. This family was previously described as active on 3-sulfate-N-acetylglucosamine, a linkage that was never described in O-glycans. In a similar way, the authors forgot to include the subfamily S1_4 in their analysis.
- In figure 1A, the schematic representation of chondroitin sulfate is incorrect (is missing the sulfated groups).

Reviewer #5 (Remarks to the Author):

The paper by Ducarmon et al describes the development of an automated tool for annotating metagenomic data with CAZyme families and predicted substrates targeted. This would indeed be a useful addition to the field as this currently can't be done with metagenomic data leading to problems with enzyme annotation and subsequent predictions of potential substrates targeted. The authors then demonstrate the utility of their tool by identifying two novel mucin users from the human gut microbiota.
My expertise isn't in bioinformatics so I couldn't install Cayman to test, but hopefully the program is easy to use as the authors indicate – all of my comments are main about some of the inferences made in relation to the glycans targeted by the human gut microbiota.
1. Fig 1A. Starch – only resistant starch is classified as dietary fibre. This should be highlighted.
2. Fiig 1B. While GAGs will be present in meat, they will also likely be in large amounts in the intestine from sloughed epithelial cells (both small and large intestine have significant normal cell turnover). This should also be noted.
3. Fig 6C. Care should be taken in assigning cellulose as a target for the human gut microbiota (see also line 430). Cellulose breakdown in the human gut is thought to be limited, esp in modern industrialised societies (see e.g. PMID: 38484069). This is due to lack of dedicated cellulose degraders, but also likely the fast transit time of the human gut vs dedicated herbivores. Cooking food can obviously influence the accessibility of cellulose, but I'm not aware of any studies where dedicated insoluble cellulose degrading apparatus has been characterised in human gut microbes. If these exist please reference the studies here.
4. Fig 6C. mannans and heteromannans. Are these plant b-mannans (cell wall or storage polysaccharides), or alpha mannans as found in the fungal cell wall? (see e.g PMID: 31649168). Does Cayman distinguish between these?
5. Fig 2c – shouldn't Akkermansia light up as a red dot in the mucin colour coded box? Seems to be only Bacteroides spp that light up red in all (DF, mucin, GAGs)?
6. Fig 4d, legend - line 268 states 'dots indicate outliers' – I can't see any dots on Fig 4d?
7. Lines 373-376 states that Akkermansia is the principal carrier of GT31 in HIS, but not LMIS individuals. From Cazy GT31 appears to be only found in Akkermansia (in bacteria). So is GT31 lacking in LMIS or carried by another taxa? Or is it because Akkermansia are rare in LMIS individuals? (see e.g. PMID: 31649168).
8. Lines 466-468 – I don't think can claim that the analysis presented here 'revealed' points ii and iii – both are well known features of CBM-GH associations.
Typo – line 544 'better' written twice

****

Version 3:

Reviewer comments:

Reviewer #4

(Remarks to the Author)

(Remarks on code availability)

Decision Letter:

Our ref: NMICROBIOL-23123479C

30th January 2026

Dear Georg,

Thank you for submitting your revised manuscript "Large-scale computational analyses of gut microbial CAZyme repertoires enabled by Cayman" (NMICROBIOL-23123479C). It has now been seen by one of the original referees who are happy with the code. Therefore we'll be happy in principle to publish it in Nature Microbiology, pending minor revisions to satisfy the referees' final requests and to comply with our editorial and formatting guidelines.

If the current version of your manuscript is in a PDF format, please email us a copy of the file in an editable format (Microsoft Word or LaTex)-- we can not proceed with PDFs at this stage.

We are now performing detailed checks on your paper and will send you a checklist detailing our editorial and formatting requirements in about a week. Please do not upload the final materials and make any revisions until you receive this additional information from us.

Thank you again for your interest in Nature Microbiology Please do not hesitate to contact me if you have any questions.

Sincerely,

Version 4:

Decision Letter:

10th March 2026

Dear Georg,

I am pleased to accept your Resource "Cayman enables large-scale analysis of gut microbiome CAZyme repertoires" for publication in Nature Microbiology. Thank you for having chosen to submit your work to us and many congratulations.

Over the next few weeks, your paper will be copyedited to ensure that it conforms to Nature Microbiology style. We look particularly carefully at the titles of all papers to ensure that they are relatively brief and understandable.

Once your paper is typeset, you will receive an email with a link to choose the appropriate publishing options for your paper and our Author Services team will be in touch regarding any additional information that may be required. Once your paper has been scheduled for online publication, the Nature press office will be in touch to confirm the details.

You may wish to make your media relations office aware of your accepted publication, in case they consider it appropriate to organize some internal or external publicity. Once your paper has been scheduled you will receive an email confirming the publication details. This is normally 3-4 working days in advance of publication. If you need additional notice of the date and time of publication, please let the production team know when you receive the proof of your article to ensure there is sufficient time to coordinate. Further information on our embargo policies can be found here:
https://www.nature.com/authors/policies/embargo.html

After the grant of rights is completed, you will receive a link to your electronic proof via email with a request to make any corrections within 48 hours. If, when you receive your proof, you cannot meet this deadline, please inform us at rjsproduction@springernature.com immediately. You will not receive your proofs until the publishing agreement has been received through our system

Due to the importance of these deadlines, we ask you please us know now whether you will be difficult to contact over the next

month. If this is the case, we ask you provide us with the contact information (email, phone and fax) of someone who will be able to check the proofs on your behalf, and who will be available to address any last-minute problems.

Acceptance of your manuscript is conditional on all authors' agreement with our publication policies (see https://www.nature.com/nmicrobiol/editorial-policies). In particular your manuscript must not be published elsewhere.

Authors may need to take specific actions to achieve compliance with funder and institutional open access mandates. If your research is supported by a funder that requires immediate open access (e.g. according to Plan S principles or the NIH public access policy) then you should select the gold OA route, and we will direct you to the compliant route where possible. Because authors warrant under our subscription licensing terms that they haven't committed to licensing any version of their article under a licence inconsistent with the terms of our agreement – including the applicable embargo period – publication under the subscription model isn't suitable for authors whose funders require no embargo.

If you have any questions about our publishing options, costs, Open Access requirements, or our legal forms, please contact ASJournals@springernature.com

An online order form for reprints of your paper is available at https://www.nature.com/reprints/author-reprints.html. All co-authors, authors' institutions and authors' funding agencies can order reprints using the form appropriate to their geographical region.

We welcome the submission of potential cover material (including a short caption of around 40 words) related to your manuscript; suggestions should be sent to Nature Microbiology as electronic files (the image should be 300 dpi at 210 x 297 mm in either TIFF or JPEG format). Please note that such pictures should be selected more for their aesthetic appeal than for their scientific content, and that colour images work better than black and white or grayscale images. Please do not try to design a cover with the Nature Microbiology logo etc., and please do not submit composites of images related to your work. I am sure you will understand that we cannot make any promise as to whether any of your suggestions might be selected for the cover of the journal.

You can now use a single sign-on for all your accounts, view the status of all your manuscript submissions and reviews, access usage statistics for your published articles and download a record of your refereeing activity for the Nature journals.

To assist our authors in disseminating their research to the broader community, our SharedIt initiative provides you with a unique shareable link that will allow anyone (with or without a subscription) to read the published article. Recipients of the link with a subscription will also be able to download and print the PDF.

As soon as your article is published, you will receive an automated email with your shareable link.

With kind regards,

P.S. Click on the following link if you would like to recommend Nature Microbiology to your librarian http://www.nature.com/subscriptions/recommend.html#forms

** Visit the Springer Nature Editorial and Publishing website at www.springernature.com/editorial-and-publishing-jobs for more information about our career opportunities. If you have any questions please click here.**

Open Access This Peer Review File is licensed under a Creative Commons Attribution 4.0 International License, which permits use, sharing, adaptation, distribution and reproduction in any medium or format, as long as you give appropriate credit to the original author(s) and the source, provide a link to the Creative Commons license, and indicate if changes were made.
In cases where reviewers are anonymous, credit should be given to 'Anonymous Referee' and the source.
The images or other third party material in this Peer Review File are included in the article's Creative Commons license, unless indicated otherwise in a credit line to the material. If material is not included in the article's Creative Commons license and your intended use is not permitted by statutory regulation or exceeds the permitted use, you will need to obtain permission directly from the copyright holder.
To view a copy of this license, visit https://creativecommons.org/licenses/by/4.0/

Reviewer #1 (Remarks to the Author):

Ducarmon et al.

Large scale computational analyses of gut microbial CAZyme repertoires enabled by Cayman

Carbohydrate-active enzymes (CAZymes) are an important group of enzymes in the human gut microbiome involved in oligo- and polysaccharide metabolism of both plant and host origin. Given that humans almost completely lack the ability to metabolise such glycans, and given that the altered microbiome functions are associated with various diseases, bioinformatic analysis tools to detail out CAZyme composition in given metagenomic datasets will continue to be valuable for the field. One important existing resource, that is updated regularly, is CAZy (www.CAZy.org), which describes which enzymes target which substrates. However, to infer substrates from large metagenomics datasets and to expedite reporting of CAZyme compositions would require development of additional bioinformatics tools. Thus, the authors developed a scalable command line tool (Cayman), with the addition of a manually curated database of CAZyme substrates, which would streamline the process of detecting biologically meaningful CAZyme shifts in the gut microbiome metagenomic datasets. No new data were generated and the authors used existing metagenomic datasets from public repositories to test their tool. Overall, this is an interesting concept. The authors used their tool and substrate annotation: 1) to explore the overall CAZyme repertoires of gut bacteria using The Genome Taxonomy Database (GDTB) database; and 2) to examine the CAZyme repertoire in the context of Western and non-Western diet and colorectal cancer. The methods and analysis are easy to follow. However, there are a number of concerns that challenge the novelty and broader applicability of this study, which altogether dampen the overall enthusiasm about this study. See seven major reservations about this study outlined below.

NB: with regards to the assessment of the scalability, and ease to use Cayman, hopefully, this can be better judged by bioinformatics specialists (other reviewers).

Hierachical substrate annotation:

1) The authors note in the abstract that "interpretation of substrate preference is lacking". The recent publication of DBCan3 in May 2023, which authors properly cite, contains a manually curated database of CAZYme substrates on the CAZyme subfamily level (link to the sheets: https://bcb.unl.edu/dbCAN2/download/Databases/dbCAN-sub.substrate.mapping.xls), which are surprisingly similar efforts compared to the current approach of the authors. Additionally, the dbCAN3 curated table contains a higher grouping of mucin and host-glycan (although groupings of dietary fibers are missing). Did the authors use this table as a resource during substrate annotation? What is the overlap between their annotation and one in dbCAN3? dbCAN3 is updated annually and is well maintained. How do authors plan to address this practical point? Moreover, how does the usage of dbCAN2 version 2020 relate to making the Cayman tool up to date? How many CAZymes have been added to the database in the meantime? Finally, dbCAN3 provides a substrate prediction tool. Could this be incorporated with the Cayman to extend the scope of analysis to less

studied species and increase the overall knowledge of substrate specificity in downstream analysis?

Response: We thank the reviewer for this relevant comment. First, dbCAN3 and Cayman were independent and parallel efforts and the dbCAN3 substrate list was not directly used for the Cayman substrate annotations in the original submission. Our substrate curation started in early 2021, when the dbCAN substrate list was not yet available. Nevertheless, we have performed a careful and comprehensive comparison between dbCAN3's and Cayman's substrate annotations for this revision. This was done on 281 CAZyme families and resulted in high agreement. Of note, Cayman substrate annotations are subfamily-resolved, while dbCAN3 annotations are only done at the CAZyme family level, thereby potentially missing out on important substrate interpretation differences between subfamilies. In this comparison, we also found a few instances where dbCAN3 substrate annotations could be used to improve Cayman annotations. We have updated all our results and figures with these improved substrate annotations.

Regarding the substrate prediction approach by dbCAN3 and its utility in annotating understudied species, we agree with the reviewer that substrate prediction tools will be needed and we have addressed this (see "Action taken").

Concerning the scope of dbCAN2/3 relative to Cayman, 69 'overall' CAZyme families were newly added to the latest dbCAN release, representing an increase of 15.9% of CAZyme families relative to the 'freeze' of our database. We agree with the reviewer that it will be important to regularly update Cayman and we foresee that the first update of the tool will come within a year (we therefore also versioned all of our software and substrate tables).

Action taken: We have added Supplementary Figure 1, which displays how well the 281 CAZyme family substrate annotations agree between Cayman and dbCAN3. We have also added this into the text in lines 112-114. We have additionally stated in lines 143-146 that we foresee Cayman to be updated in the coming year and that it is broadly applicable to microbial communities of various environments.

To make Cayman's functionality easily usable for this substrate prediction, we included an option in our software tool (https://github.com/zellerlab/cayman subcommand annotate_proteome) to predict both CAZyme domains and corresponding substrates of novel sequence sets.

2) Although authors justify their hierarchical grouping of CAZymes as potentially biologically meaningful based on a publication (reference 17), cases presented in the publication only use group 1 hierarchy (glycan substrate grouping). Since the authors did not provide any use cases Group 2 and Group 3 functional annotation, the overall biological utility of such classification cannot be judged. Therefore, biological relevance and the claim that "These can be used for interpretation of downstream statistical analysis at various levels of granularity" (lines 111-112) cannot be fully confirmed based on the presented analysis. Additionally, due to the lack of experimental knowledge, many CAZymes entries from Groups 2 and 3 are unknown, which limits the usability of such a system of annotation at the current state. This also raises a question about the timely maintenance of Cayman.

Response: We agree with the reviewer that lower-level substrate annotations were featured insufficiently.

Action taken: We have added a figure panel to the main manuscript (Fig. 6c) which displays enrichments/depletions across all three substrate hierarchies for the CRC meta-analysis. This for instance shows that in CRC patients there is a significant depletion of cellulose and hemicellulose-metabolising CAZymes and a significant enrichment of glycoprotein-metabolising CAZymes (agreeing with the enrichment of mucin-metabolising CAZymes). See lines 428-431.

We have additionally generated the same plot for the Low/middle income setting vs High income setting (LMIS/HIS; previously called Western/non-Western) meta-analysis and included this as panel Extended Data Fig. 6d and added additional main text describing these findings (lines 298-308).

Lastly, we now also illustrate in lines 118-123 the different levels of hierarchies and examples thereof.

Exploration of CAZymes in human gut microbiomes:

3) By leveraging Cayman, the authors confirm a previously described capacity of glycan degradation in Bacteroides and Parabacteroides genera, while highlighting Hungatella and Eisenbergiella genera from Firmicutes phylum, as Firmicutes genera are particularly rich in CAZyme repertoire. However, the sentence "but to our knowledge, neither genus has been experimentally studied for their glycan utilization capabilities“ (lines 147-149) is not completely true. Rawat et al. (doi: 10.1128/aem.01546-22) studied the growth of 4 glycosaminoglycans of an isolate of Hungatella Hathweyi. Interestingly, in the same publication, in Figure 1 by using a similar approach (HHMER with dbCAN2), they report an abundance of GAG Cazymes in Eisenbergiella spp, as well as in some other Firmicutes.

Response: The reviewer is correct that the statement “but to our knowledge, neither genus has been experimentally studied for their glycan utilization capabilities” is inaccurate.

Actions taken: We have corrected the statement, moved it to the Discussion and properly referenced Rawat et al. in line 501. We would however like to clarify that mucin-metabolisation capacity has not been reported, neither for *Hungatella* nor for *Eisenbergiella*. In the revised manuscript, we have included new experimental data for mucin utilisation by *Hungatella* and *Eisenbergiella* (see our responses below for details as well as new Fig. 3 and Extended Data Figs 3 and 4). Text describing this can be found in lines 232-246.

4) Moreover, substrate specificity analysis outlined Eisenbergiella and Coprobacter as promising mucin degraders. An experimental validation of such findings derived from Cayman is essential, as this would serve as a proof- of-concept for the genetic prediction of substrate annotation curated by the authors. For example, growing the type strains of both isolates on minimal media with purified mucin O-glycans and showing with transcriptomic analysis that indeed the identified CAZymes are involved in mucin degradation. Without such validation, the ambiguity in labelling a strain as a mucin degrader would not be pacified.

Response: We agree with the reviewer that such experimental validations are important. When attempting to perform the suggested experiments, we realised that this was less straightforward than anticipated, because minimal media did not support the growth of these gut bacteria (see Extended Data Fig. 3 in the revised manuscript). We therefore ended up performing experiments with rich media supplemented (or not as a control) with mucin and report in the revised manuscript growth differences as well as transcriptional changes. Since the reviewer suggested targeting *Coprobacter*, we initially also experimented with *Coprobacter secundus*, but were unable to achieve sufficient, reproducible differential growth in presence of mucin in any of the tested conditions. Hence we focused experimental validation on *Hungatella* and *Eisenbergiella*.

Actions taken: We included results of improved growth with mucin supplementation as well as differentially expressed mucin-related CAZymes (inferred via RNA-seq) for *Hungatella hathewayi* and *Eisenbergiella tayi* (see section "Experimental validation of mucin-utilisation potential by *H. hathewayi* and *E. tayi*", lines 230-246) and added new figures (Fig. 3 and Extended Data Fig. 3 and 4 in the revised manuscript).

5) Lastly, the claim "Taken together, our analysis gives a systematic overview of the carbohydrate-related metabolic potential of human gut bacteria " (lines 177-179) needs to be backed up by more in-depth analysis. We agree with the authors that their analysis is more comprehensive compared to previous reports (10.1038/nrmicro3050) and we generally find Figure 2a quite a useful overview of CAZyme abundance and broad substrate specificity. To add additional value to the overview, the species-level diversity in the CAZyme repertoire should be explored (possibly as extended data figure(s) and a table that could be used as a resource). As an illustrative example of why such information is necessary: just by looking at Figure 2, Ruminococcus genus is not enriched in CAZyme repertoire for mucin degradation but we know that Ruminococcus gnavus (DOI: 10.1093/femsre/fuad014) and Rumonococcus torques (DOIs: 10.1101/2024.01.15.575725, 10.1172/JCI111795) are prominent mucin degraders in human gut. This existing literature raises an important question that a genus-level analysis might obscure potential diversity in the overall CAZyme richness as well as diversity in substrate specificity within the genus (i.e. on a species level). Therefore, showing which species contribute most CAZyme enriches would provide a more in-depth systematic overview that could be used as a resource by other researchers.

Response: We do agree with the reviewer that species level resolution is generally more informative than genus-level resolution and that at genus level interesting species traits could be masked.

Action taken: We have therefore supplied a large table with all species-CAZyme annotations (https://zenodo.org/records/10473258) as a resource, which provides genome-level CAZyme annotations as well as taxonomic information, allowing aggregation of CAZyme numbers at all desired taxonomic levels. We have added this information into the "Data Availability" section (lines 835-837).

Furthermore, we provide high-level CAZyme diversity overview display items in a remade Figure 2b, which is a species-level ordination of CAZyme repertoire. Additionally, a species-level zoom-in on the genomic mucin-degradation capability of select genera is included in the new Figure 3a. Due to space constraints in the main figure and as suggested by the reviewer, we have added Extended Data Fig. 2 for a more exhaustive list of bacterial species potentially involved in mucin degradation, including various *Ruminococcus* species, and their potential mucin-metabolising CAZymes. As a side note, *R. torques*, *R. gnavus* and *R. bromii* do not belong to the same genus (according to the taxonomy that we have been using), and are therefore not tightly grouped in this heatmap, but indeed our analyses also show *R. torques*/*gnavus* to be distinctly more rich in mucin-degrading genes than *R. bromii*.

6) Importantly, we would like to point out that there is striking, yet underexplored strain level diversity in glycan degrading capabilities (especially mucin and glycosaminoglycan) in some species, as experimentally proven in Pudlo et al and Porras et al. (correctly referenced by authors at 7 & 20). As an illustrative example, Bacteroidetes ovatus strain has high diversity in the capability of growth on mucin-o-glycans (see Pudlo et al). This has to be addressed in the discussion of the results and the overall utility of genetic-phenotype prediction.

Response: We agree with the reviewer that there is probably strong and undescribed strain level diversity in glycan-degrading capabilities.

Action taken: To facilitate future investigations into strain-level diversity, we have supplied a large table with all genome-level CAZyme annotations (https://zenodo.org/records/10473258) as a resource, allowing aggregation of CAZyme numbers at all desired taxonomic levels (including sub-species and strain-level). We have added this information into the “Data Availability” statement (lines 835-837).

To emphasize the important point of strain-level diversity to the readers, we have included this aspect into the Discussion with a reference to Pudlo et al., as suggested by the reviewer (lines 490-493).

CAZyme capability in the context of Western and non-wester diets and colorectal cancer:

7) The analysis revealed a surprising finding of an interesting high abundance of CAZyme in the Western diet and enriched in mucin and GAG Cazymes. An additional analysis properly excluded the possibility that the finding is solely emerging from the difference in reading mappings. The authors properly addressed the potential reasons for these findings in the discussion. However, the authors need to take on board the comments made above regarding Exploration of CAZymes in human gut microbiomes.

Response: As already acknowledged above, the reviewer rightly points out that, by analyzing CAZyme repertoires in the context of Westernisation (which we now rephrased to high-income settings (HIS) and low-middle income settings (LMIS)) and colorectal cancer on the genus-level, there is the possibility that we miss more fine-grained stratification in lower taxonomic levels.

Action taken:

We have made changes to the Discussion reflecting this observation by the reviewer, see lines 488-493 and 520-522.

Minor point:

Line (111-113) – The example of CBM75 is not backed up in figure 1A as referenced and might confuse the reader.

Response: We thank the reviewer for this comment.

Action taken: We have deleted the reference to Figure 1a from the text to avoid confusion. Additionally, we have replaced the CBM75 example, as this is a very small family, with CBM2, a much more widespread family. We think this is a better, more easily accessible example. The corresponding textual changes can be found in lines 118-123.

Reviewer #2 (Remarks to the Author):

This is a large body of work and a significant analysis of the existing microbial data. The correlations it brings out are incredibly interesting and are of value to a broad community not just CAZymologists. It would have been nice, however, if even just some simple wet experiments had been done to prove the utility of the software, i.e. growing suspected O-glycan degraders on O-glycans. This is a simple experiment that could be conducted in collaboration if the authors did not have the facilities.

Response: We thank the reviewer for recognizing the importance of our work and agree on the importance of experimental validations of our computational findings.

Action taken: We have added experimental work in an entirely new section in the manuscript ("Experimental validation of mucin-utilisation potential by *H. hathewayi* and *E. tayi*", lines 230-246) and added a new figure (Fig. 3 in the revised manuscript, as well as Extended Data Figs. 3 and 4). Our experiments indeed show that growth of both *H. hatheway* and *E. tayi* is increased and accelerated in the presence of mucin (most clearly shown in Fig. 3b). See also comment 4 by reviewer 1 and our response there.

I think the potential impact of the Cayman needs to be brought out more in the introduction and how it exceeds the limitations of what is currently available. It was not immediately clear to the size in the step the authors were claiming. It did seem to come through more in the discussion.

Response: The key contribution of this work is that it makes available to the community the ability to quickly and conveniently profile CAZyme abundances in metagenomes. Together with the CAZyme substrate annotations we provide, this facilitates CAZyme-centric metagenomic studies and subsequent inferences on substrate utilisation.

Action taken: To highlight Cayman's unique capabilities and how it exceeds the limitations of other tools, we have added lines 81-83 and lines 86 to the Introduction.

To further strengthen the resource aspect of our work, we now also provide free and open-access CAZyme annotations of >100,000 microbial genomes via Zenodo. We have added this information to the "Data Availability" statement (lines 835-837).

Additionally, we added more information on the tool and its capabilities to the Results in lines 143-146 and to the Discussion in lines 474-475.

Lastly, to more clearly illustrate the differences and similarities between Cayman and dbCAN3, we provide a conceptual comparison of both tools as Table S3.

There needs to be more careful analysis of the literature and what the CAZymes actually do and what is known about their biochemistry. I have made specific comments about GH29 and GH95, and GHs used in Figure 2B to imply mucin foraging. There is also no analysis of carbohydrate sulfatases which have been shown to be important for host glycan metabolism.

Response: We thank the reviewer for bringing our attention to these important aspects. We are commenting on the issue regarding GH29/GH95 in our responses further below and will here focus on carbohydrate sulfatases.

In order to obtain sulfatase annotations for our genomes, we leveraged the SulfAtlas tool (https://academic.oup.com/nar/article/51/D1/D647/6786190). Unfortunately, this tool does not allow for large-scale analysis: The reason for this is that the HMMs and relevant cut-offs are not publicly available (and were not made available to us by the authors after personal inquiry). Instead, only a manual web interface is provided, which is limiting file upload to a maximum of 50Mb size, thereby prohibiting annotation of a our gut gene catalog with ~14 million genes.

As this precluded a comprehensive annotation of all our genomes, we focused on obtaining sulfatase annotations manually for all of the *Eisenbergiella* (n=53) and *Hungatella* (n=66) genomes we included in our work. Additionally, we included 50 randomly chosen *Akkermansia* genomes, given that this genus is well-established as a prolific mucin metaboliser. We then selected the 5 sulfatase subfamilies that target sulfate residues from colonic mucin (S1_11, S1_15, S1_16, S1_20 and S1_46). These families were also selected based upon consultation with the SulfAtlas authors and previous research (e.g. https://www.nature.com/articles/s41589-022-01039-x).

Action taken: The results of our investigation of the occurrences of these 5 mucin-targeting sulfatases in the genomes of these 3 genera were included as Supplementary Figure 2. We describe these results in the revised manuscript in lines 197-201.

All in all the work looks strong and is interesting but is a large number of very interesting correlations that need to be phenotypically tested. Maybe the power is in the software can be accessed and deployed by wet labs, but how easy that will be for novices I am unsure, but the work is clearly of value.

Response: We thank the reviewer for the supportive stance of our work. In our view the main contribution of our work is the metagenomic profiler we developed (Cayman), which allows users to directly profile their metagenomic reads and get CAZyme abundances and substrate enrichments as output, which facilitates CAZyme-centric studies both genomically as well as on a metagenomic level. The Cayman software is available through various repositories now and is easily deployed (we have already had contact/collaborations with various labs who successfully employed the software). Motivated by this comment we have taken the opportunity to revise our manuscript to further clarify these aspects.

Action taken: As mentioned earlier in this rebuttal letter (e.g. comment 4 by reviewer 1), we have added experimental work to this paper that is shown in the new main manuscript Figure 3 and Extended Data Figs 3 and 4. With regard to the unique strong points of our work, we would furthermore like to refer to (i) Cayman being the first freely available (and easy-to-use) software tool capable of profiling CAZymes from metagenomes (as we have now clarified in lines 81-83 and line 86, lines 143-146 and lines 474-475) and (ii) resource availability on Zenodo of >100k microbial genomes and their CAZyme annotations, as we have now explicitly added under the "Data Availability" statement in lines 835-837.

Specific comments

Lines 67-71

CAzy is a database cataloguing the literature so the limitations here are clear but dbCAN can annotate genomes and predict active as it says in the header on the webage, what limitation does dbCAN have? Is it only one genome at a time? Can it not handle metagenomic data? There is also no mention of PULDB which is a useful resource for predicting enzyme function/substrate target by PUL composition. Is the issue these are not open source?

Response: As mentioned in our response above, the key contribution of this work is that it makes available to the community the ability to quickly and conveniently profile CAZyme abundances in metagenomes - which dbCAN does not. We have highlighted these differences in Table S3. dbCAN can handle more than one genome at a time (should one download the HMMs and apply them locally), but it indeed is not intended to be used on metagenomic data. Furthermore, while PULDB is a very useful resource, it is not publically available as a software tool and can therefore not easily be leveraged for data-driven, large-scale (meta-)genomic surveys as we have conducted here.

Action taken: We have created a new Table S3 that highlights the differences and commonalities between Cayman and dbCAN3. We have furthermore added lines 81-83 to the Introduction to highlight that Cayman is the first open-source available metagenomic profiler.

Line 78-86

The issue I have here is the phrase 'CAZyme families into higher-level, biologically meaningful substrate groups' this must be done using data from the literature and CAZy,

how else will Cayman predict what these enzymes are doing? When you have a PUL it can be easy to attempt to infer the target but when there is no PUL organisation in an organism simply ascribing its substrate preferences based on CAZymes that are distally located on the genome is a risky business, especially if the software is used by non CAZymologists.

Response: To clarify, essentially our tool indeed incorporates literature/CAZy information to both annotate substrates to CAZy families (through curation of substrate data from literature/CAZy), and – by annotating novel gene sequences to CAZy families via sequence similarity searches – to annotate substrates to gene sequences. Our tool is not systematically utilizing genomic co-localization (as a PUL predictor would) to infer CAZy family membership or substrates.

Action taken: We have adapted our wording in lines 112-114 and added lines 121-123.

Line 99 Developing and curating a CAZyme substrate scheme

How is the example of CBM75 helpful? There is only 1 sequence on CAZy and the CBM is appended to GH43_16 which are arabinofuranosidases, xylosidases, and xylanases.

Response: We agree that CBM75 was perhaps not the best chosen example. We have therefore replaced this with CBM2, a much more common and relevant CBM.

Action taken: We have replaced CBM75 with CBM2 as an example (see lines 118-123).

Line146-147

Among these, Hungatella and Eisenbergiella stood out as distinctly rich in CAZymes (with 224 genes from 78 families and 404 genes from 106 families, respectively),

This is a remarkable finding but it would be good to see these genes listed somewhere in the supplemental. Saying the Bacteroides have high numbers and the Firmicutes (now Bacillota) have less is well known in the field. The data that two Bacillota organisms, that have not been well studied, have huge numbers needs more explicit data than just a bar on the bar chart if others are to use the data as a springboard.

Response: We thank the reviewer, this is a good suggestion.

Action taken: We have supplied a table in the Zenodo repository (https://zenodo.org/records/10473258) that lists genome-level CAZyme annotations including, but not limited to, *Hungatella* and *Eisenbergiella*, which could serve as a starting point for the interested reader. We have added information about the availability of these annotations under the "Data Availability" statement (lines 835-837).

Figure 2B

I think there needs to some caution about calling all the CAZyme family selected as mucin related:

GH2 is a polyspecific family working on a vast number of substrates both host and plant

Response: This is indeed a helpful remark. We had indeed annotated GH2 as well as many other CAZyme families accordingly as polyspecific families (i.e. as families with more than one substrate) in our (original) substrate annotation scheme, but this was not clear in the text.

Action taken: We are now stating more clearly in the manuscript that many CAZyme families are polyspecific in terms of substrates and that we also annotated them as such (lines 121-123).

GH31 and GH35: These both have members working on plant and host glycans has this been accounted for?

Response: Yes, we agree with the reviewer that GH31 and GH35 are examples of such polyspecific CAZymes. This information as captured by our annotations is included in Table S1. We would like to point out that this information was already encoded in the original submission (column A "Origin" contains information on the host origin of a given CAZyme in Table S1). With regard to GH31 and GH35, we had indeed captured that these CAZymes can both derive from, and act on, glycans from various organisms.

GH29 and GH95: within both these families the presence of Thr or His in the active site correlates with if the enzymes are L-fucosidases or L-galactosidases. L-galactose is no found in mucin and any sequences with targeting this would need to be removed. In addition, plant glycans also contain L-fucose (refs: https://doi.org/10.1038/ncomms8481 and doi: 10.1038/s41589-022-00983-y_

Response: We understand the reviewer and the mentioned publications to essentially suggest that specific variants of these enzymes might act as L-galactosidases (as opposed to their canonical activity of L-fucosidases).

We had a close look at both publications and examined the evidence of this functional dichotomy. We note 2 main take-aways: Firstly, both papers provide *anecdotal* evidence for the substrate differences and, crucially, in both papers they disproved their respective hypothesis (of AA variants dictating the functional shift between L-fucosidase and L-galactosidase) with mutant enzymes:

Study by Rogowski et al. (2016, Nature Communications): "The importance of the **Thr370** in **BACOVA_03438** is demonstrated by the complete loss of activity of the T370H mutant against CX [This is Corn Xylan] (Supplementary Table 7). While the **Thr/His polymorphism** [In reference to the GH95 of *B. bifidum*] may be a specificity determinant for galactosidase and fucosidase activity within GH95, **the paucity of enzymes characterized in this family prevents a definitive conclusion regarding its functional significance**. Indeed, the observation that the **T370H mutation does not increase fucosidase activity** suggests that L-Gal to L-Fuc specificity is driven by more than just the −1 site (Supplementary Table 7)." (Emphasis from us)

Study by Robb et al. (2022, Nature Chemical Biology): “However, notable differences between GH29 fucosidases and BpGH29 are a serine or glycine in place of His189 and a tyrosine residue at the position of Ser187 (Fig. 2f). **Despite retaining its thermal stability, a S187Y/H189S double mutant of BpGH29 failed to *display significant activity on either pNP-α-l-Fuc or pNP-α-l-Gal,* pointing to unknown subtleties in the active site architectures of these enzymes that impart their selectivity.”** (Emphasis from us)

Furthermore, since they reason from a structural perspective, querying and interpreting these differences in thousands of gene sequences within the scope of our study would pose a substantial computational challenge. Given these complications, we feel that it is beyond the scope of this paper to attempt to resolve these dichotomies in our substrate annotations.

Action taken: We adapted the Discussion to highlight the subtleties of functional inference in light of the 2 linked papers. See lines 488-490.

GH84 is infamous as being an O-GlcNAcase removing GlcNAc from phosphorylation sites.

Response: This is correct and had also been captured in our original substrate annotation scheme as such: GlcNAc was annotated as one of the main targeted substrates (Table S1, row 334).

GH108 the only annotation on CAZy is lysozyme? This would mean it targets peptidoglycans?

Response: This is indeed correctly observed by the reviewer and we are grateful to the reviewer for pointing this out.

Action taken: We have corrected the substrate annotation of GH108 (Table S1) into peptidoglycan, as we also noted that this was one of the few families where we had substrate disagreement with dbCAN3 (where peptidoglycan was indeed correctly annotated).

GH130 has members specific for fungal beta mannann

Response: This is correct and our substrate annotation scheme (Table S1) had this information already encoded in the original submission.

Lines 167-174

The inferences are nice, and make great hypotheses, but it would have been better to show that some of these organisms are specialist mucin degraders. At the moment this is a correlation and not proof.

Response: This is a valid point, and similar to comments of other reviewers. To address this, we performed differential growth assays in the presence/absence of mucin as well as differential gene expression analysis.

Action taken: We included results of improved growth with mucin supplementation as well as differentially expressed mucin-related CAZymes (inferred via RNA-seq) for *Hungatella hathewayi* and *Eisenbergiella tayi* (see section "Experimental validation of mucin-utilisation potential by *H. hathewayi* and *E. tayi*", lines 230-246) and added new figures (Fig. 3 b, c and Extended Data Fig. 3 and 4 in the revised manuscript). See also comment 4 by reviewer 1 and our response there.

Line 173

genomic copies for several mucin-targeting GHs, including α-fucosidases (GH29 and GH95),

The absence of presence of Thr or His in the base of the -1 subsite needs checking, and L-galactosidases discounted.

Response: This connects to a previous comment about AA variants correlating with a functional dichotomy between L-fucosidases and L-galactosidases conditional on single AA differences. We have provided a detailed answer to this comment above.

Line 261

On the other hand, the most Western-enriched CAZyme, CBM58, is part of the SusG protein

strictly speaking CBMs are not CAZymes (Carbohydrate active enzymes), but I am not sure if there is a catch all term alternative that covers CBMs (SGBPs are also not CAZymes).

Response: The reviewer is right that CBM58, as any other CBM, should not be labelled CAZyme since it is not 'carbohydrate-active'.

Action taken: We would argue that for the sake of both internal consistency, as well as consistency with language used by the original CAZy developers, it is justifiable to leave this as is. However, given that we agree with the reviewer that this terminology can be confusing, we have added a sentence to the Introduction that explicitly states that CBMs are technically not CAZymes (see line 77).

Figure 4 D again it would be interesting to see the polymorphism spread in the GH95s and how this maps to the data.

Response: We have addressed this point in our response to the reviewer's comment above that started with the phrase "GH29 and GH95: within both these families the presence of Thr or His in the active site correlates with if the enzymes are L-fucosidases or L-galactosidases.".

Line 306 is Fig 4D at the start right as everything is about figure 4E and F.

Response: This was correctly observed by the reviewer.

Action taken: We ensured that all sub-panels of Figure 4B-G (now Figure 5) are now correctly cited.

Line 314 Fig 4F call out should be 4G

Response: We thank the reviewer for spotting this error.

Action taken: We have fixed this in the revised manuscript.

Line 365-370

Taken together, these results showed a clear enrichment of

CAZymes involved in host- and animal glycan metabolism in CRC metagenomes

I would if the authors considered using sulfatases as an important CAZyme? They are essential enzymes for the degradation of sulfated host glycans in the colon (GAGs and MUC2), and would provide stronger inferences to their data.

Response: While we agree with the reviewer about the importance of sulfatases (see our previous extensive comment to an earlier point raised by this reviewer regarding sulfatases), SulfAtlas unfortunately did not allow for large-scale annotations of genomic data with sulfatases, thereby preventing us from annotating our human gut microbial gene catalog with sulfatase information. We even had correspondence with the SulfAtlas authors regarding this point, but they were not willing to share their HMMs and relevant cut-offs with us, which we would need to conduct this analysis on our gene catalog. Therefore, we could unfortunately not analyse the sulfatase abundances at the metagenomic level.

Action taken: While we could not integrate the sulfatase analyses at the large-scale metagenomic level, we have included sulfatase analyses at the genome level of the newly discovered mucin metabolisers (*E. tayi* and *H. hathewayi*), as well as of *Akkermansia* (see Supplementary Fig. 2 and Lines 197-201).

Lines 388-400

CBMs often mirror the activity of the enzyme they are appended too but not always, sometimes they bind an abundant glycan that is close to the target of the enzyme. Plant cell wall degrading xylanases have been shown to have cellulose binding CBMs.

The ‘guilt by association’ is not novel to this paper and known CBMs appended to large genes of unknown function have been used as probes to find new enzymes functions previously.

Response: The reviewer correctly points out that the guilt by association approach is not novel.

Action taken: To ensure this detail is not lost on the reader, we updated the Results in lines 451-452. Moreover, given the justified point of the reviewer that CBMs within enzymes (e.g.

xylanases and cellulose-binding CBMs) can sometimes bind multiple substrates, we have added lines 458-461 and added the reference of Herve et al., PNAS 2010. Of note, we have removed our example of guilt-by-association substrate assignment of mucin for CBM51, as CBM51 has mucin as annotation in our revised substrate scheme (it was unannotated in our original substrate scheme). Instead, we now showcase an example of what the reviewer rightly pointed out here, namely that CBM62 can potentially bind various substrates (lines 461-462)

Reviewer #3 (Remarks to the Author):

Summary -

In this manuscript, the authors describe their new Cayman tool for profiling CAZymes and annotating their preferred substrates in metagenomic data. Their hierarchical substrate annotation scheme will enable researchers to more confidently assign functions to specific CAZymes. The authors deploy their tool to look for differences in CAZyme substrate preferences between industrialized and non-industrialized populations and between patients with colorectal cancer and healthy patients. The results of these analyses are intuitive, and provide confidence in the tool. Overall, the tool is useful, the methodology is appropriate, the figures are informative, and the manuscript is well written. I commend the authors on a great tool and a great manuscript. I don't have any major concerns with the work. Below I provide some minor comments to help improve the presentation.

Response: We thank the reviewer for the positive remarks about our manuscript.

Specific Comments -

The Western/Non-Western dichotomy is Euro-centrist language, which is falling more and more out of fashion. I often find it confusing when countries like Japan, Australia, and South Korea are labeled with 'Western' lifestyles. It seems more precise to use dichotomies like: Developed/Developing, Industrialized/Non-industrialized, Urban/Rural, and High vs. Low and Middle Income. To avoid sounding dated in a few years, I suggest using more precise language, when it comes to the exact populations included in your first meta-analysis.

Response: We have extensively discussed this issue and agree with the reviewer that this may come across as eurocentric even though it seems to be the prevailing terminology used in the field.

Actions taken: We have replaced all “Western” and “non-Western” labels with “High-income setting (HIS)” and “Low-middle income setting (LMIS)”. We believe this more accurately reflects the populations and we hope this addresses the concerns raised by the reviewer. For detailed information on each study/population included in our meta-analysis, we’d like to refer the reviewer to Table S4.

I often found myself getting lost in the acronyms, and certain acronyms didn't appear to be defined (dbCAN, pHMMs). Please double check that you've defined them, and perhaps make a little table that summarizes these acronyms for the reader.

Action taken: We have ensured that all acronyms used in the revised manuscript are properly introduced.

line 24: Maybe change 'richer' to 'enriched'?

Action taken: We have adopted this suggestion.

line 27: Suggest changing 'deprived of' to 'depleted in'

Action taken: We have adopted this suggestion.

line 60: suggest replacing 'the stark contrast has been instrumental to uncover shifts of gut' to 'the contrast has uncovered shifts in key'

Action taken: We have adopted this suggestion.

line 567: change 'on based' to 'based on'

Action taken: We have adopted this suggestion.

Response to Reviewer Comments on **Large-scale computational analyses of gut microbial CAZyme repertoires enabled by Cayman** by Q. R. Ducarmon, N Karcher et al.

Reviewer #1 (Remarks to the Author):

I thank the authors for their revisions; the manuscript is substantially improved. Cayman will be an important resource for the community.

Minor point: Line 544 word "better" duplicated

Response: We thank the reviewer for the constructive comments and are glad they share our view that Cayman will be an important resource for the community.

Action taken: We have removed the duplication of 'better' in what is now line 458.

Reviewer #4:

As the previous reviewer 2 mention in is previously revision, the tool developed to predict the specificity of CAZymes is of scientific interest but this should be also address carefully if this is to be used by non CAZYmologists. Of relevance, the reviewer asked if the authors leverage the previous knowledge regarding the substrate recognition of GH95s to which the authors apparently dismiss as 'anecdoctal evidence for substrate differences'. In my opinion this is quite a misjudgment of wording regarding a previously published article (PMID: 26112186) where structural and biochemical data backs up the conclusions. The lack of understanding of such concepts by the authors raises questions regarding how this software can be applied to distinguish between activities in poly-specific families.

Response: We agree with the reviewer that there was sufficient structural and biochemical data in the referenced paper to substantiate the point that a single amino-acid difference (Thr vs His) in the active site justifies calling a GH95-annotated protein an L-Galactosidase rather than an L-Fucosidase. However, the authors themselves also noted that this single amino acid difference does not necessarily explain the substrate specificity. Nonetheless, we are well aware that in general subtle amino acid changes can sometimes profoundly alter enzyme function (and surely many such cases are yet to be discovered), which poses a challenge for computational inference of gene/domain functionality, whether it be CAZymes or other functions.

Action taken: We have acknowledged the general limitations of computational functional inference based on sequence homology in the Discussion. As this also

relates to a similar issue observed for GH95 example, we now discuss both cases in conjunction (lines 420-426):
"However, our analysis also shares limitations with this and other studies: CAZyme and substrate annotations are transferred via sequence similarity. All such approaches tend to miss functional consequences (e.g. substrate changes) of subtle differences in protein sequence and are unaware of context, i.e. function is ascribed considering each gene in isolation."

Additionally, both reviewer 1 and 2 request a experimental validation Hungatella and Eisenbergiella grow on mucin (here I would like to know that reviewer 1 make a point to suggest the growth on purified mucins. Although the authors grew the bacteria in a source of mucin, it has been established by now that commercially available gastric mucins are not a pure substrate and it is not similar to colonic mucins (PMID: 38947523). The commercially available mucins are highly contaminated with other sources of glycans (such as glycosaminoglycans) so the use of this substrate does not validate the claim that the bacteria grows on mucin.

Response: We agree with the reviewer that commercially available mucins (usually porcine gastric mucins) are not pure substrates and are molecularly different from human colonic mucins. However, we would argue that our experiments nonetheless provide strong evidence for mucin utilisation for the following reasons:
1) We show that our *Eisenbergiella tayi* and *Hungatella hathewayi* grow better when mucin is supplemented to a *rich* medium, not that these bacteria grow on a minimal medium with mucin as the sole carbon source. In the context of rich media, we believe mucin impurities to be less of an issue. Because *Eisenbergiella* and *Hungatella* (as many other gut bacteria) are not easily grown in minimal media, we had to pursue this as a feasible experimental validation approach.
2) Even the classic paper that described the discovery of *Akkermansia muciniphila* to be a mucin-forager was based on the usage of gastric porcine mucin (10.1099/ijs.0.02873-0) and we therefore believe that this source of mucin can still be valuable for discovering novel mucin foragers.
3) Gastric porcine mucin remains a commonly used substrate even nowadays to perform high-throughput screening of gut microbes in order to discover potential novel mucin foragers, see e.g. https://www.nature.com/articles/s41564-018-0123-9.

Neverthelesse, we do agree with the reviewer that (although not easily feasible) the cleaner experimental setting would be using minimal media with purified mucins as we now acknowledge in the revised manuscript.

Action taken: We have added the following sentence to the Discussion on line 439-442: "However, it should be noted that our experiments used (impure) gastric porcine mucin[47]. To definitively establish these bacteria as human colonic mucin foragers, showing growth on a minimal medium with purified human colonic mucin as the sole carbon source would be desirable."

Additional comments that require to be corrected:
- The reviewer 2 comment of the lack of analysis of bacterial sulfatases active on mucin. Although the authors introduced this information in the current manuscript the inclusion of the analysis of S1_46 sulfates as a family active on O-glycans is incorrect. This family was previously described as active on 3-sulfate-N-acetylglucosamine, a linkage that was never described in O-glycans. In a similar way, the authors forgot to include the subfamily S1_4 in their analysis.

Response: We had (naively) relied on the annotations provided by SulfAtlas tool and thank the reviewer for pointing this out. After double-checking the linkages targeted by S1_4 and S1_46, we agree with the reviewer that S1_4 should be included and S1_46 should be excluded.

Action taken: We have updated the text in line 175-177 to include the correct families and updated Supplementary Figure 2 to contain the correct families.

- In figure 1A, the schematic representation of chondroitin sulfate is incorrect (is missing the sulfated groups).

Response: We thank the reviewer for pointing this out and we have now included the sulfated groups into the figure.

Action taken: The figure has been adapted to include the sulfated groups.

Reviewer #5 (Remarks to the Author):

The paper by Ducarmon et al describes the development of an automated tool for annotating metagenomic data with CAZyme families and predicted substrates targeted. This would indeed be a useful addition to the field as this currently can't be done with metagenomic data leading to problems with enzyme annotation and subsequent predictions of potential substrates targeted. The authors then demonstrate the utility of their tool by identifying two novel mucin users from the human gut microbiota.
My expertise isn't in bioinformatics so I couldn't install Cayman to test, but hopefully the program is easy to use as the authors indicate – all of my comments are main about some of the inferences made in relation to the glycans targeted by the human gut microbiota.
1. Fig 1A. Starch – only resistant starch is classified as dietary fibre. This should be highlighted.

Response: We agree with this point and have adapted the name in the figure into "Resistant starch"

Action taken: We have adapted Figure 1A and changed "Starch" into "Resistant starch"

2. Fiig 1B. While GAGs will be present in meat, they will also likely be in large amounts in the intestine from sloughed epithelial cells (both small and large intestine have significant normal cell turnover). This should also be noted.

Response: This is a good point which indeed needed to be clarified.

Action taken: We have added this information into line 260-262 as follows: "Similarly, HIS metagenomes have a higher abundance ratio of CAZymes targeting GAGs (animal-derived glycans, sources for intestinal GAGs include shed epithelial cells and dietary intake) relative to those targeting dietary fibre (**Fig. 4d**)."

3. Fig 6C. Care should be taken in assigning cellulose as a target for the human gut microbiota (see also line 430). Cellulose breakdown in the human gut is thought to be limited, esp in modern industrialised societies (see e.g. PMID: 38484069). This is due to lack of dedicated cellulose degraders, but also likely the fast transit time of the human gut vs dedicated herbivores. Cooking food can obviously influence the accessibility of cellulose, but I'm not aware of any studies where dedicated insoluble cellulose degrading apparatus has been characterised in human gut microbes. If these exist please reference the studies here.

Response: We fully agree with the reviewer that this is a delicate topic. That is why we had formulated it as "CAZymes involved in cellulose and hemicellulose metabolism", which is similar to the phrasing in the referenced paper by the reviewer (PMID: 38484069), e.g. "For example, dockerin-containing GH families 2, 97, and 141 were only present in the *R. flavefaciens* rumen-associated strains and absent from the human- and NHP-gut *R. hominiciens* and *R. primaciens* genomes. These enzymes encode various hemicellulolytic activities such as mannosidase, glucoamylase, and xylanase activities, thus attesting to the richer spectrum of polysaccharides that exists in the rumen environment. Similarly, GH families coding for enzymes acting on cellulose (GH3 and GH9)".
We hope that with our phrasing we maintained the nuance that specific CAZymes are contained in these microbiomes that can target (hemi)cellulose, but that this does not necessarily mean that the complete pathway for metabolising an entire cellulose molecule is present.

Action taken: To clarify the limitations of our approach here, we have added the following text into the discussion lines 420-426, using cellulose as an example: "However, our analysis also shares limitations with this and other studies: CAZyme and substrate annotations are transferred via sequence similarity. All such approaches tend to miss functional consequences (e.g. substrate changes) of subtle

differences in protein sequence and are unaware of context, i.e. function is ascribed considering each gene in isolation. These limitations make it challenging to infer functions which require multiple enzymes, such as cellulose degradation, for which it remains unclear if performed by HIS gut microbiota at all."

4. Fig 6C. mannans and heteromannans. Are these plant b-mannans (cell wall or storage polysaccharides), or alpha mannans as found in the fungal cell wall? (see e.g PMID: 31649168). Does Cayman distinguish between these?

Response: This is something that arises from the hierarchical substrate annotation scheme we created, as Fig 6C shows analyses at the relatively high hierarchical levels. We acknowledge that in a way this is an imperfection of such a hierarchical substrate scheme. In the analysis shown both alpha and beta-mannans are grouped. We fully agree with the reviewer though that it may potentially be important to know which CAZymes are beta-mannan targeting versus alpha-mannan targeting, which is why in our hierarchical substrate scheme (Table S1) we have an additional column "H / Activity Description" wherein this even more detailed substrate information can be tracked. So while in the figure we do not make the distinction, Cayman does allow for doing so if desired.

5. Fig 2c – shouldn't Akkermansia light up as a red dot in the mucin colour coded box? Seems to be only Bacteroides spp that light up red in all (DF, mucin, GAGs)?

Response: We have double-checked and *Akkermansia* is indeed colored red in the mucin panel, but this was not easy to see since other points were partially overlapping with the one corresponding to *Akkermansia*.

Action taken: To make this clearer we have put a black circle around the 4 highlighted species (*B. uniformis, A. muciniphila, E. tayi* and *H. hathewayi*) across all panels in Figure 2c.

6. Fig 4d, legend - line 268 states 'dots indicate outliers' – I can't see any dots on Fig 4d?

Response: We thank the reviewer for bringing this mistake to our attention, this was a remnant from a previous submission.

Action taken: We have removed this line from the legend.

7. Lines 373-376 states that Akkermansia is the principal carrier of GT31 in HIS, but not LMIS individuals. From Cazy GT31 appears to be only found in Akkermansia (in bacteria). So is GT31 lacking in LMIS or carried by another taxa? Or is it because Akkermansia are rare in LMIS individuals? (see e.g. PMID: 31649168).

Response: This is an interesting observation by the reviewer. We have further looked into it and realized, based on taxonomically annotating the genes in our gene catalog that belong to GT31, that quite a few of these originate from *Blastocystis*. We have therefore profiled *Blastocystis* in all of our samples and indeed found that in the LMIS populations, GT31 partially associates with *Blastocystis* abundance (less so in the HIS populations).

Action taken: We have modified the main text in lines 331-334 and added **Figure S3**. "In contrast, in LMIS individuals, the single-celled parasite *Blastocystis* (whose genome also encodes GT31) is more strongly associated with GT31 abundance than *Akkermansia* (**Supplementary Fig. 3**)."

8. Lines 466-468 – I don't think can claim that the analysis presented here 'revealed' points ii and iii – both are well known features of CBM-GH associations.
Typo – line 544 'better' written twice

Response: Regarding lines 466-468 (now lines 408-410), we agree with the reviewer and have adapted our wording. We have additionally corrected the typo in line 458.

Action taken: Lines 408-410 have been reworded as follows:
"Taken together, this co-occurrence analysis revealed interesting conservation of CAZyme domain pairs and suggests that substrate specificity of CAZyme domains could be predicted more broadly using a 'guilt-by-association' approach[41]."
